# Fast Protein Loop Sampling and Structure Prediction Using Distance-Guided Sequential Chain-Growth Monte Carlo Method

**DOI:** 10.1371/journal.pcbi.1003539

**Published:** 2014-04-24

**Authors:** Ke Tang, Jinfeng Zhang, Jie Liang

**Affiliations:** 1 Department of Bioengineering, University of Illinois at Chicago, Chicago, Illinois, United States of America; 2 Department of Statistics, Florida State University, Tallahassee, Florida, United States of America; Fox Chase Cancer Center, United States of America

## Abstract

Loops in proteins are flexible regions connecting regular secondary structures. They are often involved in protein functions through interacting with other molecules. The irregularity and flexibility of loops make their structures difficult to determine experimentally and challenging to model computationally. Conformation sampling and energy evaluation are the two key components in loop modeling. We have developed a new method for loop conformation sampling and prediction based on a chain growth sequential Monte Carlo sampling strategy, called Distance-guided Sequential chain-Growth Monte Carlo (DiSGro). With an energy function designed specifically for loops, our method can efficiently generate high quality loop conformations with low energy that are enriched with near-native loop structures. The average minimum global backbone RMSD for 1,000 conformations of 12-residue loops is 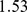 Å, with a lowest energy RMSD of 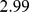 Å, and an average ensemble RMSD of 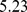 Å. A novel geometric criterion is applied to speed up calculations. The computational cost of generating 1,000 conformations for each of the x loops in a benchmark dataset is only about 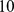 cpu minutes for 12-residue loops, compared to *ca* 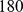 cpu minutes using the FALCm method. Test results on benchmark datasets show that DiSGro performs comparably or better than previous successful methods, while requiring far less computing time. DiSGro is especially effective in modeling longer loops (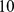
–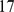 residues).

## Author Summary

Loops in proteins are flexible regions connecting regular secondary structures. They are often involved in protein functions through interacting with other molecules. The irregularity and flexibility of loops make their structures difficult to determine experimentally and challenging to model computationally. Despite significant progress made in the past in loop modeling, current methods still cannot generate near-native loop conformations rapidly. In this study, we develop a fast chain-growth method for loop modeling, called Distance-guided Sequential chain-Growth Monte Carlo (DiSGro), to efficiently generate high quality near-native loop conformations. The generated loops can be used directly for downstream applications or as candidates for further refinement.

This is a *PLOS Computational Biology* Methods article.

## Introduction

Protein loops connect regular secondary structures and are flexible regions on protein surface. They often play important functional roles in recognition and binding of small molecules or other proteins [1]–[3]. The flexibility and irregularity of loops make their structures difficult to resolve experimentally [4]. They are also challenging to model computationally [5], [6]. Prediction of loop conformations is an important problem and has received considerable attention [5]–[27].

Among existing methods for loop prediction, template-free methods build loop structures *de novo* through conformational search [5]–[7], [9], [10], [13], [14], [17], [18], [21], [23], [28]. Template-based methods build loops by using loop fragments extracted from known protein structures in the Protein Data Bank [11], [19], [27]. Recent advances in template-free loop modeling have enabled prediction of structures of long loops with impressive accuracy when crystal contacts or protein family specific information such as that of GPCR family is taken into account [14], [23], [25].

Loop modeling can be considered as a miniaturized protein folding problem. However, several factors make it much more challenging than folding small peptides. First, a loop conformation needs to connect two fixed ends with desired bond lengths and angles [8], [12]. Generating quality loop conformations satisfying this geometric constraint is nontrivial. Second, the complex interactions between atoms in a loop and those in its surrounding make the energy landscape around near-native loop conformations quite rugged. Water molecules, which are often implicitly modeled in most loop sampling methods, may contribute significantly to the energetics of loops. Hydrogen bonding networks around loops are usually more complex and difficult to model than those in regular secondary structures. Third, since loops are located on the surface of proteins, conformational entropy may also play more prominent roles in the stability of near-native loop conformations [29], [30]. Approaches based on energy optimization, which ignore backbone and/or side chain conformational entropies, may be biased toward those overly compact non-native structures. Despite extensive studies in the past and significant progress made in recent years, both conformational sampling and energy evaluation remain challenging problems, especially for long loops (*e.g.*, 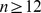).

In this paper, we propose a novel method for loop sampling, called Distance-guided Sequential chain-Growth Monte Carlo (DiSGro). Based on the principle of chain growth [15], [31], [32], [34], [35], the strategy of sampling through sequentially growing protein chains allows efficient exploration of conformational space [15], [34]–[37]. For example, the Fragment Regrowth via Energy-guided Sequential Sampling (FRESS) method outperformed previous methods on folding benchmark HP sequences [15], [33]. In addition to HP model [15], sequential chain-growth sampling has been used to study protein packing and void formation [35], side chain entropy [29], [38], near-native protein structure sampling [30], conformation sampling from contact maps [39], reconstruction of transition state ensemble of protein folding [40], RNA loop entropy calculation [37], and structure prediction of pseudo-knotted RNA molecules [41].

In this study, we first derive empirical distributions of end-to-end distances of loops of different lengths, as well as empirical distributions of backbone dihedral angles of different residue types from a loop database constructed from known protein structures. An empirical distance guidance function is then employed to bias the growth of loop fragments towards the 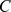-terminal end of the loop. The backbone dihedral angle distributions are used to sample energetically favorable dihedral angles, which lead to improved exploration of low energy loop conformations. Computational cost is reduced by excluding atoms from energy calculation using REsidue-residue Distance Cutoff and ELLipsoid criterion, called Redcell. Sampled loop conformations, all free of steric clashes, can be scored and ranked efficiently using an atom-based distance-dependent empirical potential function specifically designed for loops.

Our paper is organized as follows. We first present results for structure prediction using five different test data sets. We show that DiSGro has significant advantages in generating native-like loops. Accurate loops can be constructed by using DiSGro combined with a specifically designed atom-based distance-dependent empirical potential function. Our method is also computationally more efficient compared to previous methods [8], [9], [18], [22], [42]. We describe our model and the DISGRO sampling method in detail at the end.

## Results

### Test set

We use five data sets as our test sets. Test Set 1 contains 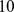 loops at lengths four, eight, and twelve, for a total of 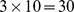 loops from 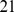 PDB structures, which were described in Table 2 of zRef. [8]. Test Set 2 consists of 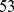 eight, 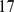 eleven, and 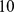 twelve-residue loops from Table C1 of Ref. [42]. Several loop structures were removed as they were nine-residue loops but mislabeled as eight-residue loops: (1awd, 55–63; 1byb, 246–254; and 1ptf, 10–18). Altogether, there are 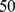 eight-residue loops. Test Set 3 is a subset of that of [5], which was used in the RAPPER and FALCm studies [10], [22]. Details of this set can be found in the “Fiser Benchmark Set” section of Ref. [10]. Test Set 4 is taken from Table A1–A6 of Ref. [42]. Test Set 5 contains 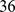 fourteen, 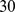 fifteen, 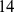 sixteen and 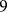 seventeen-residue loops from Table 3 of Ref. [23]. Test Set 1 and 2 are used for testing the capability of DiSGro and other methods in generating native-like loops. Test Set 3, 4, and 5 are used for assessing the accuracy of predicted loops based on selection from energy evaluation using our atom-based distance-dependent empirical potential function. Our results are reported as global backbone RMSD, calculated using the N, 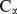, C and O atoms of the backbone.

### Loop sampling

To evaluate our method for producing native-like loop conformations, we use Test Set 1 and 2.

We generate 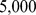 loops for each of the 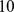 loop structures in Test Set 1 at length 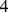, 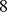, and 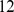 residues, respectively. We compare our results with those obtained by CCD [8], CSJD [12], SOS [18], and FALCm [22]. The minimum RMSD among 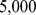 sampled loops generated by DiSGro are listed in Table 1, along with results from the four other methods.

**Table 1:** Minimum backbone RMSD values of the loops sampled by five different algorithms.

Length	Loop	CCD	CSJD	SOS	FALCm	DiSGro
12-res	1cruA_358	2.54	2.00	2.39	2.07	1.84
	1ctqA_26	2.49	1.86	2.54	1.66	1.36
	1d4oA_88	2.33	1.60	2.44	0.82	1.50
	1d8wA_46	4.83	2.94	2.17	2.09	1.17
	1ds1A_282	3.04	3.10	2.33	2.10	1.82
	1dysA_291	2.48	3.04	2.08	1.67	1.45
	1eguA_508	2.14	2.82	2.36	1.71	2.13
	1f74A_11	2.72	1.53	2.23	1.44	1.46
	1qlwA_31	3.38	2.32	1.73	2.20	0.79
	1qopA_178	4.57	2.18	2.21	2.36	1.77
	Average	3.05	2.34	2.25	1.81	**1.53**
8-res	1cruA_85	1.75	0.99	1.48	0.62	1.34
	1ctqA_144	1.34	0.96	1.37	0.56	0.70
	1d8wA_334	1.51	0.37	1.18	0.96	0.93
	1ds1A_20	1.58	1.30	0.93	0.73	0.62
	1gk8A_122	1.68	1.29	0.96	0.62	1.08
	1i0hA_145	1.35	0.36	1.37	0.74	0.80
	1ixh_106	1.61	2.36	1.21	0.57	0.39
	1lam_420	1.60	0.83	0.90	0.66	0.63
	1qopB_14	1.85	0.69	1.24	0.92	0.87
	3chbD_51	1.66	0.96	1.23	1.03	0.67
	Average	1.59	1.01	1.19	0.72	**0.80**
4-res	1dvjA_20	0.61	0.38	0.23	0.39	0.31
	1dysA_47	0.68	0.37	0.16	0.20	0.09
	1eguA_404	0.68	0.36	0.16	0.22	0.39
	1ej0A_74	0.34	0.21	0.16	0.15	0.09
	1i0hA_123	0.62	0.26	0.22	0.17	0.13
	1id0A_405	0.67	0.72	0.33	0.19	0.33
	1qnrA_195	0.49	0.39	0.32	0.23	0.19
	1qopA_44	0.63	0.61	0.13	0.30	0.39
	1tca_95	0.39	0.28	0.15	0.09	0.11
	1thfD_121	0.50	0.36	0.11	0.21	0.05
	Average	0.56	0.40	0.20	0.22	**0.21**

Minimum backbone RMSD values of the loops sampled by CCD, CSJD, SOS, FALCm and DiSGro for different loop structures. CCD result was obtained from Table 2 of Ref. [8]. CSJD result was obtained from Table 1 of Ref. [12]. SOS result was obtained from Table 1 of Ref. [18]. FALCm result was obtained from Table 2 of Ref. [22].

Accurate loops of longer length are more difficult to generate. For loops with 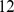 residues, DiSGro generates more accurate loops than other methods. Our method has a mean of 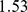 Å for the minimum RMSD, compared to 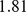 Å for FALCm, the next best method in the group [22]. The minimum RMSD of nine of the ten 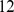-residue loops have RMSD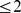 Å, while five loops of the ten generated by FALCm have RMSD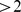 Å. Compared to the CCD, CSJD, and SOS methods, our loops have significantly smaller minimum RMSD (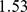 Å *vs* 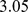, 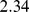, and 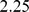 Å, respectively, Table 1). The average minimum global backbone RMSD for 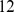-residue loops can be further improved when we increase the sample size of generated loop conformations. The minimum global RMSD is improved to 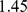 Å, 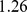 Å, and 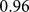 Å when the sample size is increased to 20,000, 100,000, and 1,000,000, respectively. Further improvement would likely require flexible bond lengths and angles.

For loops with 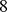 residues, DiSGro has an average minimum RMSD value smaller than the CCD, CSJD, and SOS methods (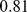 Å *vs* 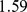 Å, 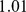 Å, and 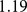 Å, respectively, Table 1). In eight of the ten 8-residue loops, DiSGro achieves sub-angstrom accuracy (RMSD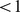 Å), although the mean of minimum RMSD of 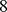-residue loops is slightly larger than that from FALCm (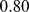 Å *vs* 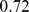 Å).

For loops with 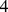-residue, the mean of the minimum RMSD (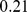 Å) by DiSGro is significantly smaller than those by the CSJD and the CCD methods (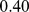 Å and 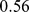 Å, respectively), and is similar to those by the SOS and FALCm methods(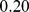 Å and 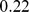 Å, respectively). Noticeably, three of the ten loops have RMSD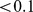 Å, indicating our sampling method has good accuracy for short loop modeling.

These loops can be generated rapidly. The computing time per conformation averaged over 5,000 conformations for 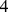, 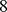, and 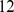-residues is 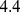, 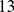, and 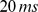 using a single AMD Opteron processor of 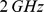. In addition to improved average minimum RMSD, DiSGro seems to take less time than CCD (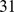, 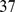, and 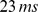 on an AMD 1800+ MP processor for the 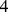, 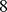, and 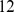-residue loops), and is as efficient as SOS (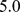, 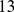, and 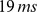 for the 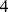, 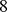, and 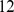-residue loops on an AMD 1800+ MP processor).

Reducing the number of trial states in DiSGro can further reduce the computing time, with some trade-off in sampling accuracy. For example, when we take 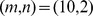, the computing time per conformation averaged over 5,000 conformations for 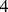, 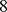, and 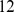-residues is only 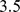, 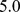, and 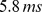, respectively, with the average minimum RMSDs comparable to those from SOS's (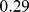 Å *vs* 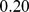 Å, 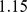 Å *vs* 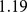 Å, and 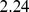 Å *vs* 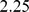 Å for the 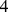, 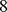, and 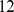-residue loops, respectively). Although the CSJD loop closure method has faster computing time (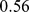, 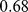, and 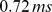 on AMD 1800+ MP processor), the speed of DiSGro is adequate in practical applications.

We compare DiSGro in generating near-native loops with Wriggling [43], Random Tweak [44], Direct Tweak [42], [45], 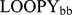 [45], and PLOP-build [13] using Test Set 2. The minimum RMSD among 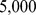 loops generated by DiSGro are listed in Table 2, along with results from the other methods obtained from Table 2 in Ref. [42]. Direct Tweak and 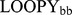 from the LoopBuilder method and our DiSGro have better accuracy in sampling than Wriggling, Random Tweak, and PLOP-build methods. For loops with 11 and 12-residues, these three methods are the only ones that can generate near-native loop structures with minimal RMSD values below 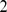 Å. Among these, DiSGro outperforms 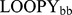 in generating loops at all three lengths: the average minimal RMSD (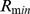) is 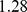 Å *vs.* 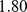 Å for length 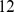, 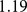 Å *vs.* 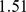 Å for length 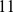, and 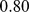 Å *vs.* 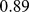 Å for length 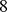, respectively. Compared to the Direct Tweak sampling method, DiSGro has improved 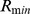 for 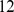-residue loops (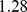 Å *vs* 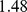 Å), slightly improved 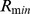 for 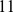-residue loops (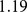 Å *vs* 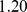 Å) and inferior 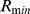 for 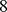-residue loops (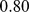 Å *vs* 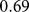 Å). Overall, these results show that DiSGro are very effective in sampling near-native loop conformations, especially when modeling longer loops of length 11 and 12.

**Table 2:** Comparison of 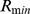 of the loop conformations sampled by DiSGro and six other methods using Test Set 2 used by Ref. [42].

	Average minimum backbone RMSD (*R*_m*in*_)						
Length	Random Tweak	CCD	Wriggling	PLOP-build	Direct Tweak	LOOPY_bb_	DiSGro
8	1.22	1.20	1.43	0.99	0.69	0.89	**0.80**
11	2.22	2.11	2.24	2.18	1.20	1.51	**1.19**
12	2.64	2.57	2.68	2.69	1.48	1.80	**1.28**

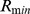
 denote the average minimum backbone RMSD of the loop ensemble. Random Tweak, CCD, Wriggling, PLOP-build, Direct Tweak and 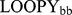 results were obtained from Table 2 of Ref. [42].

Our DiSGro method can generate accurate loops and has significant advantages for longer loops compared to previous methods. Using RMSD values calculated from three backbone atoms N, 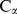, and C for all loop lengths lead to the same conclusion.

### Loop structure prediction and energy evaluation

To assess the accuracy of loops selected by our specifically designed atom-based distance-dependent empirical potential function, we test DiSGro using Test Set 3 and follow the approach of reference [22] for ease of comparison. Because of the high content of secondary structures, these loops are very challenging to model. In the study of [22], 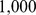 backbone conformations with the best scores evaluated by DFIRE potential function [46] were retained after screening 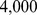 generated backbone conformations for each loop. Loop closure and steric clash removal were not enforced to the 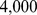 conformations. We follow the same procedure, except the DFIRE potential function is replaced by our atom-based distance-dependent empirical potential function. The ensemble of the selected 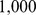 backbone conformations are then subjected to the procedure of side-chain construction as described in the Section “Side-chain modeling and steric clash removal”. The loop conformations with full side-chains are then scored and ranked by the atom-based distance-dependent empirical potential function. Our results are summarized in Table 3.

**Table 3:** Comparison of 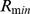, 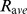 and 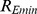 of the lowest energy conformations of the loops sampled by RAPPER, FALCm4 and DiSGro using Test Set 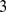.

Length	# of Targets	RAPPER			FALCm			DiSGro		
		*R*_m*in*_	*R*_a*ve*_	*R_Emin_*	*R*_m*in*_	*R*_a*ve*_	*R_Emin_*	*R*_m*in*_	*R*_a*ve*_	*R_Emin_*
4	35	0.43	1.65	0.86	0.33	0.92	0.54	**0.21**	**0.66**	**0.48**
5	35	0.53	2.27	1.00	0.44	1.63	0.92	**0.25**	**1.11**	**0.84**
6	36	0.69	3.06	1.85	0.47	2.34	1.36	**0.44**	**1.74**	**1.22**
7	38	0.78	3.79	1.51	0.58	2.74	1.17	**0.55**	**2.23**	**1.08**
8	32	1.11	4.16	2.11	0.84	3.69	1.87	**0.80**	**2.87**	**1.72**
9	37	1.29	5.00	2.58	0.95	4.21	2.08	**0.94**	**3.64**	**1.82**
10	37	1.67	5.66	3.60	1.45	5.07	3.09	**1.15**	**3.96**	**2.33**
11	33	1.99	6.71	4.25	1.47	5.76	3.43	**1.39**	**4.96**	**2.98**
12	34	2.21	6.96	4.32	1.74	6.31	3.84	**1.53**	**5.23**	**2.99**

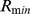
, 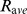 and 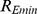 denote the average minimum backbone RMSD, the average ensemble RMSD and the average RMSD of the lowest energy conformations of the 1,000 loop ensemble with the same length, respectively.

**Figure 1:**
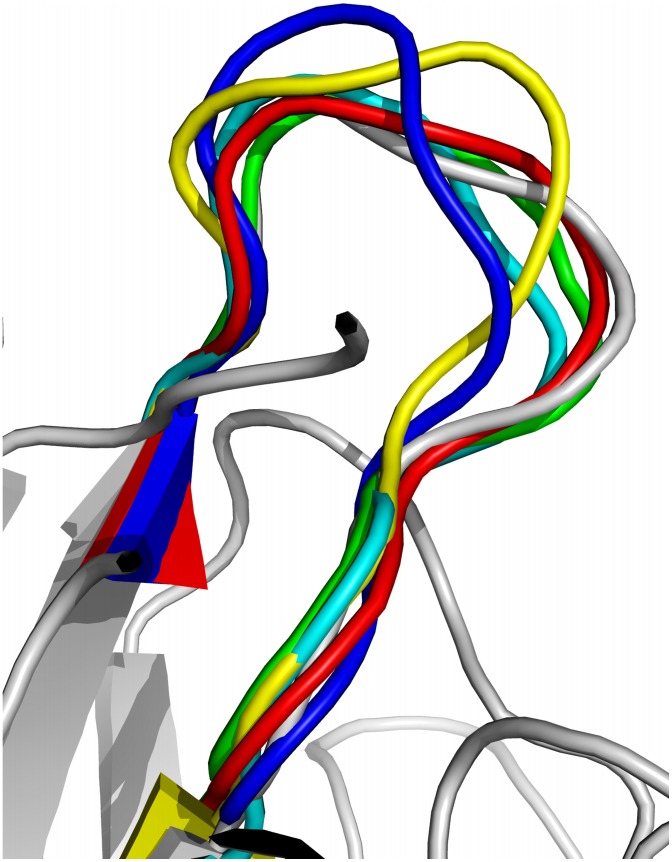
Top five lowest energy loops of length 12 for single-metal-substituted concanavalin A (pdb 1scs, residues 199–210). The lowest energy loop after side-chain construction is colored in red, and the native structure is in white.

We measure the average minimum backbone RMSD 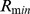, the average ensemble RMSD 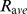, and the average RMSD of the lowest energy conformations 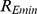 of the 1,000 loop ensemble with the same length. Overall, DiSGro performs significantly better than FALCm and RAPPER in 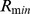, 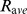 and 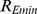 for all loop lengths. Compared to FALCm, DiSGro shows significant advantages in 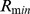 on sampling long loops of 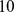
–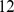 residues. Our method has 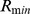 of 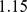 Å compared to 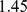 Å for 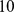-residue loops, 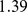 Å compared to 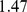 Å for 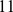-residue loops, and 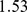 Å compared to 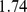 Å for 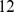-residue loops, respectively. For example, as can be seen in Figure 1, the lowest energy loop (red) of a 12-residue loop in the protein 1scs (residues 199–210) has a 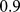 Å RMSD to the native structure (white). The generated top five lowest energy loops are all very close to the native loop, yet are diverse among themselves.

DiSGro also generates loops with smaller 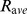 compared to FALCm in loops with length ranging from 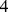 to 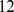, indicating DiSGro can generate ensemble of loop conformations with enriched near native conformations. Furthermore DiSGro achieves better modeling accuracy using the atom-based distance-dependent empirical potential function. Compared to FALCm, DiSGro has a 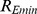 of 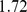 Å *vs* 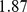 Å for 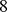-residue loops, 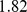 Å *vs* 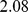 Å for 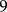-residue loops, 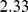 Å *vs* 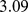 Å for 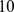-residue loops, 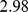 Å *vs* 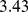 Å for 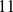-residue loops, and 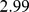 Å *vs* 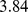 Å for 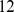-residue loops, respectively.

DiSGro is also much faster than other methods. The reported typical computational cost of FALCm is 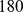 cpu minutes for 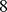
–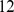 residue loops on a Linux server of a 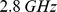 2-core Intel Xeon processor [47]. The computation cost for DiSGro method is only 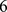 and 10 cpu minutes for 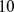 and 12–residue loops on a single 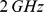 AMD Opteron processor, respectively. In addition, FALCm has a size restriction, and it only works with proteins with 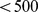 residues. In contrast, the overall protein size has no effect on the computational efficiency of DiSGro since the numbers of atoms for energy calculation that are retained by the ellipsoid criterion are bounded.

The LOOPER method is an accurate and efficient loop modeling method using a minimal conformational sampling method combined with energy minimization [17]. The test set used in the LOOPER study is the original Fiser data set without removal of any loops. Therefore, it is different from Test Set 3 used in the RAPPER and FALCm studies [10], [22]. For ease of comparison, we compare DiSGro to the LOOPER using the test set with 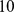
–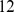-residue loops from [17]. Our results are summarized in Table 4.

**Table 4:** Comparison of accuracy of modeled loops using the original Fiser data set of loops with 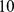
–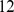 residues.

Length	Targets	DiSGro/LOOPER			
		*R*_B*kb,ave*_	*R*_B*kb,med*_	*R*_A*tm,ave*_	*R*_A*tm,med*_
10	40	**2.30**/2.66	**2.20**/2.39	**3.39**/3.58	**3.18**/3.35
11	40	**2.63**/3.35	**2.25**/2.76	**3.58**/4.30	**3.30**/3.60
12	40	**3.20**/4.08	**2.39**/3.80	**4.18**/5.22	**3.60**/4.96

The accuracy achieved by LOOPER and DiSGro at different loop length using the original Fiser data set of loops with 10–12 residues is listed. 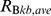, and 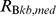 denote the mean and median of backbone RMSD, while 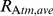, and 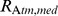 denote the mean and median of all-heavy atoms RMSD of the lowest energy conformations with the same loop length.

We denote 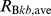 and 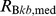 as the mean and median of backbone RMSD of the lowest energy conformations with the same loop length. Similarly, we use 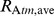, and 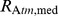 to denote the mean and median RMSD values of all-heavy atoms. DiSGro shows improved prediction accuracy compared to LOOPER in both backbone and all-heavy atom RMSD. For the 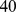 loops of length 12, 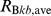 is 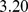 Å compared to 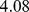 Å, while the median 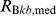 is 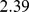 Å compared to 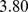 Å. It also has better all-heavy atom RMSD of 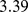 Å/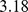 Å (mean/median), compared to 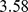 Å/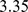 Å for 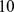-residue loops, 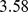 Å/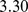 Å compared to 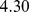 Å/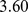 Å for 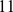-residue loops, and 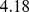 Å/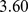 Å compared to 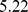 Å/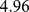 Å for 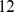-residue loops.

It is worth noting that DiSGro outperforms LOOPER in speed as well. For a loop with 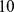 residues, the time cost of DiSGro is 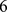 minutes using a 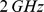 CPU versus 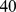 cpu minutes using a 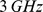 processor according to Figure 7 in the LOOPER paper [17].

Prior publications also allowed us to compare results in loop structure predictions based on energy discrimination using Test Set 4 with results obtained using the LoopBuilder method [42]. Following [42], we generated 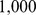 closed loop conformations for eight-residue loops, 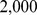 for nine-residue loops, 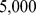 for ten, eleven, and twelve-residue loops, and 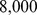 for thirteen-residue loops. Energy calculations are carried out using our atom-based distance-dependent empirical potential function. The average RMSD of the lowest energy conformations, 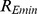, are then compared between these two methods. The results are summarized in Table 5.

**Table 5:** Comparison of 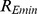 of the loop conformations sampled by Loop Builder and DiSGro using Test Set 4 taken from the Loop Builder study [42].

		Average prediction accuracy (*R_Emin_*)	
Length	# of Targets	LoopBuilder	DiSGro
8	63	1.31	**1.59**
9	56	1.88	**1.83**
10	40	1.93	**1.83**
11	54	2.50	**2.38**
12	40	2.65	**2.62**
13	40	3.74	**3.26**

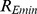
 denote the average RMSD of the lowest energy conformations of the loop ensemble. Results of LoopBuilder were obtained from Table 5 of Ref. [42].

Compared to LoopBuilder, DiSGro has better 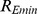: 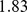 Å *vs* 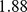 Å for 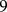-residue loops, 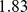 Å *vs* 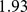 Å for 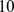-residue loops, 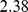 Å *vs* 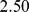 Å for 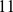-residue loops, 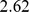 Å *vs* 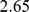 Å for 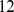-residue loops, and 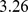 Å *vs* 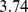 Å for 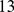-residue loops, respectively. DiSGro has inferior performance in selecting 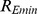 for 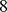-residue loops (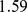 Å *vs* 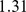 Å). The average time using LoopBuilder for twelve-residue loops was around 4.5 hours or 270 minutes, while the computational time using DiSGro is around 10 minutes. Overall, DiSGro has equal or slightly better performance than LoopBuilder in average prediction accuracy of loop structures with far less computing time.

To test the feasibility of DiSGro in modeling longer loops with length 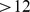, we use the Fiser 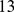-residue loops data set to generate and select low energy loop conformations. 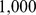 conformations with low energy are obtained. The mean of minimum backbone RMSD 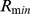 of 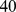 loops with 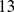-residue is 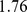 Å, and the median is 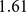 Å. The mean/median of the backbone RMSD 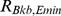, and all heavy atom RMSD 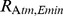 of the lowest energy conformations are 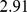 Å/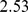 Å and 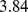 Å/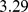 Å, respectively (Table 6).

**Table 6:** Accuracy of modeled loops by DiSGro using the original Fiser data set of loops with 13 residues.

Target	PDB	Start	End	Sequence	*R*_m*in*_	*R*_e*n,Ave*_	*R*_B*kb,Emin*_	*R*_A*tm,Emin*_
1	154l	21	33	akpeglsycgvsa	2.14	7.08	2.42	3.20
2	1aba	5	17	ygydsnihkcgpc	1.83	4.37	5.08	5.58
3	1amp	252	264	nprihttqdtlan	2.12	6.03	2.88	4.32
4	1art	160	172	yydaenhtldfda	2.67	7.28	5.62	6.80
5	1byb	139	151	vdnepifhgrtai	2.85	9.42	3.98	5.02
6	1cbn	32	44	ciiipgatcpgdy	1.50	6.34	2.12	3.28
7	1cgt	38	50	aydatcsnlklyc	2.38	6.90	4.47	4.74
8	1clc	258	270	mqypdgsgrvahk	2.25	5.17	4.45	5.72
9	1ctm	34	46	evpqavlpdtvfe	0.92	4.98	1.21	1.98
10	1fas	4	16	yshtttsrailtn	1.56	8.21	1.73	2.57
11	1fnd	47	59	kitgddapgetwh	1.41	10.25	1.51	2.02
12	1frd	37	49	lpfschsgscssc	1.56	8.78	5.83	6.46
13	1fus	91	103	thtgasgnnfvgc	2.36	6.61	3.33	4.78
14	1gof	70	82	mlprqdgnqngwi	1.17	3.21	2.66	2.95
15	1ivd	429	441	grkqetrvwwtsn	2.48	5.76	2.82	5.60
16	1l58	50	62	igrncngvitkde	1.62	12.03	2.13	3.30
17	1msc	33	45	issnsrsqaykvt	2.20	6.74	5.33	6.18
18	1osa	55	67	vdadgngtidfpe	0.82	10.27	0.82	1.35
19	1pca	204	216	ypygyktqspadk	2.49	7.25	5.99	6.30
20	1php	59	71	hlgrpkgkvveel	1.40	8.61	1.40	2.16
21	1prn	213	225	ydnglstagdqvt	1.41	5.32	1.55	1.72
22	1rec	159	171	fgkkdddklteke	1.18	9.66	1.18	2.35
23	1srp	46	58	wngykvfgqpvkl	1.02	7.58	1.90	2.24
24	1thg	493	505	dpnvgtnllqwdq	1.39	5.42	2.11	2.97
25	1thw	102	114	isnikgfnvpmdf	1.31	3.75	1.87	2.86
26	1trb	222	234	lrdtqnsdniesl	1.56	4.42	1.64	2.83
27	1xif	99	111	fkdggftandrdv	1.21	7.02	3.23	4.23
28	2ctc	204	216	ypygyttqsipdk	2.57	6.42	4.28	4.76
29	3cyr	34	46	hhlvdgkesyakc	2.18	5.89	5.62	6.17
30	2exo	51	63	tepsqnsfsfgag	2.64	7.58	2.64	3.98
31	2pia	58	70	slcndsqernryv	1.29	3.83	2.80	4.12
32	2por	170	182	idspdtalmadme	1.27	6.20	1.60	2.23
33	2sil	86	98	iyndrvnsklsrv	1.19	5.82	3.37	4.00
34	3grs	129	141	haaftsdpkptie	1.61	9.05	1.78	3.03
35	4icb	53	65	ldkngdgevsfee	1.84	9.06	2.01	2.95
36	5fx2	93	105	cgdssyeyfcgav	2.27	5.15	4.61	6.57
37	5p21	115	127	gnkcdlaartves	1.79	7.09	2.81	3.79
38	5pti	9	21	pytgpckariiry	1.15	5.66	1.42	3.07
39	7rsa	86	98	etgsskypncayk	1.84	11.38	1.84	2.73
40	8dfr	166	178	padiqeedgiqyk	1.97	10.08	2.21	2.68
Mean					**1.76**	**7.04**	**2.91**	**3.84**
Median					**1.61**	**6.82**	**2.53**	**3.29**

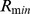
 and 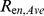 are the minimum backbone RMSD and the average backbone RMSD of the 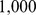 sampled conformations, respectively. 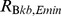 and 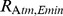 are the backbone and all heavy atoms RMSD of the lowest energy conformations in the ensemble.

With extensive conformational sampling using molecular mechanics force field, the Protein Local Optimization Program (PLOP) can predict highly accurate loops [13], [14], [23]. We tested DiSGro using Test Set 5 consisting of 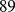 loops with length 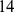
–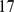 and compared results with those using PLOP. Here the sampling and scoring processes were similar to those used in Test Set 3, except 100,000 backbone conformations were generated. We measured the average minimum backbone RMSD 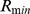 and the average RMSD of the lowest energy conformations 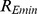. Our results are summarized in Table 7.

**Table 7:** Comparison of 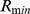, 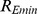 and 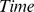 of the loop conformations sampled by PLOP and DiSGro using Test Set 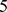.

Length	# of Targets	PLOP			DiSGro		
		*R*_m*in*_	*R_Emin_*	*Time* (hours|days)	*R*_m*in*_	*R_Emin_*	*Time* (hours)
14	36	NA	1.19	216.0|9.0	**1.58**	**3.73**	**0.73**
15	30	NA	1.55	309.6|12.9	**1.80**	**3.91**	**0.72**
16	14	NA	1.43	278.4|11.6	**1.88**	**4.16**	**0.81**
17	9	NA	2.30	408.0|17.0	**2.18**	**4.46**	**0.95**

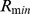
 and 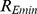 denote the average minimum backbone RMSD and the average RMSD of the lowest energy conformations of the loop ensemble.

Loops predicted by the PLOP method have smaller 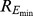 compared to DiSGro [23], although DiSGro samples well and gives small 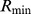 of 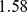 Å for 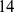-residue loops, 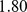 Å for 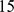-residue loops, 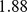 Å for 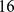-residue loops, and 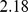 Å for 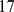-residue loops. For loops of length 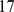, the 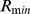 of 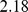 Å is less than the reported 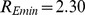 Å using PLOP, although it is unclear whether the 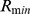 of loops generated by PLOP is less than 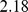 Å. Overall, DiSGro is capable of successfully generating high quality near-native long loops, up to length 17. The accuracy of 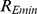 of loops generated by DiSGro may be further improved by using a more effective scoring function.

We also compared the computational costs of the two methods. The average computing time for DiSGro is 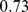, 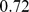, 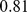, and 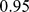 hours for loops of lengths 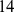, 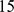, 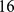, and 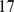 using a single core AMD Opteron processor 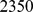, respectively, which is more than two orders of magnitude less than the time required for the PLOP method (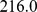, 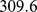, 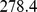, and 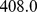 hours for loops of length 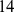, 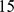, 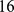, and 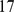 residues, respectively).

### Improvement in computational efficiency

**Figure 2:**
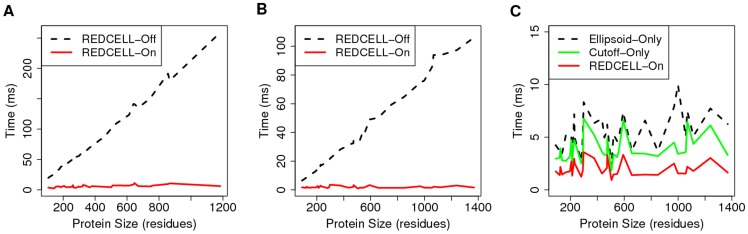
The time cost of energy calculations for generating one single loop. (A) The plot of computing time versus protein size show a large time saving of “Redcell-On” (red solid curve) compared to “Redcell-Off” (black dashed curve) for 12-residue loops, and (B) The plot of 6-residue loops. (C) Plot of computing time versus protein size show “Redcell-On” (red solid curve) has significantly improved computational time cost compared to “Ellipsoid-Only” (black dashed curve) and “Cutoff-Only” (green solid curve).

We used a REsidue-residue Distance Cutoff and ELLipsoid criterion (Redcell) to improve the computational efficiency. To assess the effectiveness of this approach, we carry out a test using a set of 140 proteins (see discussion of the tuning set in Materials and Methods). We compared the time cost of energy calculation of generating a single loop, with and without this procedure. When the procedure is applied, we only calculate the pairwise atom-atom distance energy between atoms in loop residues and other atoms within the ellipsoid. When the procedure is not applied, we calculate energy function between atoms in loop residues and all other atoms in the rest of the protein. The computational cost of energy calculations for sampling single loops with 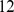 and 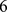-residues are shown in Figure 2A and Figure 2B, respectively.

From Figure 1, we can see that significant improvement in computational cost is achieved. The average time cost using our procedure is reduced from 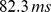 to 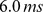 for sampling 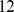-residue loops, and 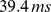 to 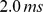 for 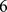-residue loops. In addition, this approach makes the time cost of energy calculations independent of the protein size (Figure 2A and Figure 2B), whereas the computing time without applying this procedure increases linearly with the protein size. The improvement is especially significant for large proteins. For example, to generate a 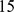-residue loop in a protein with 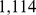 residues, the computing time is improved from 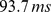 to 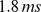, which is more than 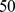-fold speed-up. Detailed examination indicates that both distance cutoff and the ellipsoid criterion contribute to the computational efficiency. Furthermore, the full Redcell procedure has improved efficiency over using either “Ellipsoid Criterion Only” or “Cutoff Criterion Only”. The computing time for generating a 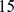-residue loops is 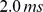 when the full Redcell procedure is applied, compared to 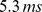, and 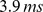, when only the ellipsoid criterion and only the distance-threshold are used, respectively (Figure 2C). Furthermore, there is no loss of accuracy in energy evaluation. Overall, Redcell improves the computational cost by excluding many atoms from collision detections and energy calculations, with significant reduction in computation time, especially for large proteins.

## Discussion

In this study, we presented a novel method Distance-guided Sequential chain-Growth Monte Carlo (DiSGro) for generating protein loop conformations and predicting loop structures. Ensembles of near-native loop conformations can be efficiently generated using the DiSGro method. DiSGro has better average minimum backbone RMSD, 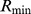, compared to other loop sampling methods. For example, 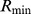 is 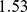 Å for 12-residue loops when using DiSGro, while the corresponding values are 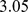 Å, 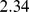 Å, 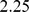 Å, and 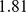 Å when using the CCD, CSJD, SOS, and the FALCm method.

DiSGro also performs well in identifying native-like conformations using atom-based distance-dependent empirical potential function. In comparison with other similar loop modeling methods, DiSGro demonstrated improved modeling accuracy, in terms of an average RMSD of the lowest energy conformations 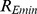 for the more challenging task of sampling longer loops of 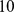
–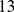 residues. For example, DiSGro outperforms FALCm [22] (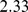 Å *vs* 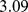 Å) and LOOPER [17] (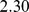 Å *vs* 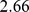 Å) in predicting 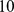-residue loops, while taking less computing time (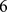 minutes *vs* 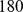 minutes for FALCm and 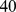 minutes for LOOPER. Compared to LoopBuilder [42], DiSGro also has better 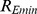: For 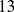-residue loops, the 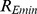 is 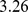 Å using DiSGro, but is 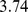 Å when using the Loop Builder. The average computing time is also faster when using DiSGro: it takes about 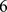 minutes to predict structures of 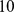-residue loops and 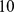 minutes for 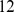-residue loops. DiSGro also works well for short loops, although this may be largely a reflection of the underlying analytical closure method [12].

There are a number of directions for further improvement. DiSGro can be further improved by adding fragments of peptides when growing loops instead of adding individual residues. Fragment-based approach has been widely used in protein structure prediction [48]–[51] and specifically in loop structure prediction [21]. It is straightforward to apply the strategy described in this study for fragment-based growth, and it will likely lead to improved sampling efficiency further and enable longer loops to be modeled. Furthermore, the energy function employed here can be further improved by optimization such as those obtained by training with challenging decoy loops using nonlinear kernel [52], and/or using rapid iterations through a physical convergence function [53], [54]. In addition, DiSGro is compatible with different loop closure methods [8], [12], [22], and experimenting with other closure strategy may also lead to further improvement.

An efficient loop sampling method such as DiSGro can help to improve overall modeling of loop structures. Currently, the hierarchical approach of the Protein Local Optimization Program (PLOP) [13], [14], [23] gives excellent accuracy in protein loop modeling, but requires significant computational time. The average time cost of modeling a 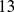-residue loop is about 4–5 days [23]. Kinematic closure (KIC) method can also make very accurate predictions of 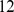-residue loops [21]. However, KIC also requires substantial computation, with about 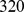 CPU hours on a single 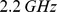 Opteron processor for predicting 12-residue loops [21]. As suggested earlier by Spassov et al [17], an efficient loop modeling method combined with energy minimization may overcome the obstacle of high computational cost. By generating high quality initial structures using DiSGro, near native conformations of loops can be used as candidates for further refinement.

## Materials and Methods

### Protein structures representation

All heavy atoms in the backbone and side chain of a protein loop are explicitly modeled. The bond lengths 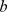 and angles 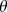 are taken from standard values specific to residue and atom type [55]. The backbone dihedral angles 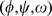 and side chain dihedral angles 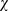 constitute all the degrees of freedom (DOFs) in our model.

### Distance-guided Sequential chain-Growth Monte Carlo (DiSGro)

In order to efficiently generate adequate number of native-like loop conformations, we have developed a Distance-guided Sequential chain-Growth Monte Carlo (DiSGro) method.

Let the loop to be modeled begins at residue 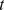 and ends at residue 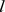. The sequence of the positions of backbone heavy atoms from 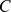 atom of residue 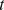 to 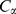 (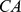) atom of residue 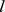 are unknown and need to be generated. We assume that the backbone atoms before and after this fragment are known. Coordinates of side chain atoms are also unknown and need to be generated if the coordinates of the 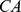 atoms they are attached to are unknown.

**Figure 3:**
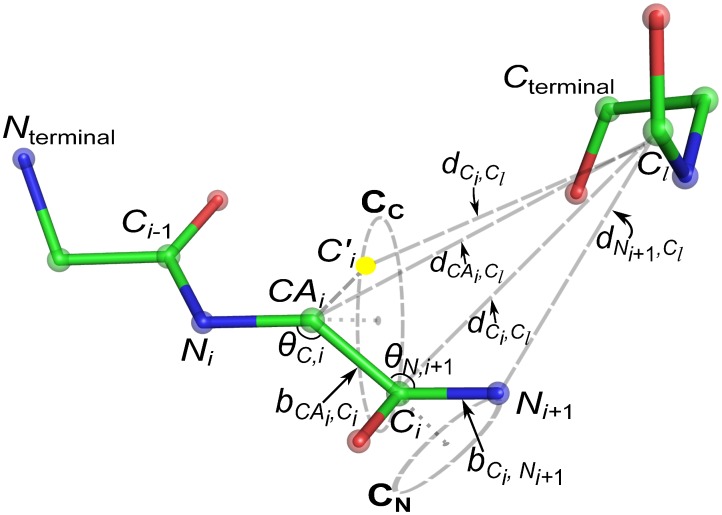
Schematic illustration of placing 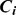 and 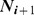 atoms. Atom 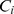 has to be on the circle 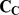. The position 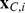 of the 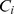 atom of residue 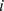 is determined by 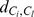, which is based on known distance 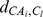 and the conditional distribution of 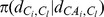. Once 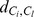 is sampled, 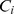 can be placed on two positions with equal probabilities. Here 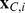 is the selected position of 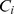. 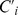 (yellow ball) is placed at the position 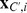 alternative to 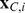. Similarly, the 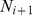 atom has to be on the circle 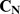 and its position 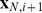 is determined by 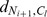 in a similar fashion.

At each step of the chain growth process, we generate three consecutive backbone atoms continuing from the backbone atom sampled at the previous step. At the 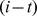-th growth step (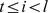), the three backbone atoms are 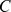 atom of residue 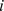, 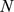 atom of residue 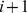, and 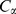 atom of residue 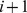 (Figure 3). The coordinates of the three atoms, 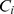, 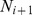 and 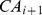, are denoted as 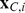, 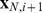, and 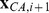, respectively. The 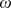 dihedral angles that determine the coordinate of 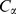 atoms are sampled from a normal distribution with mean 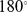 and standard deviation 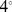. In the next section, we describe in detail in sampling of the dihedral angles 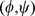, which determine the coordinates of the 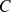 and the 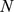 atoms.

#### Sampling backbone 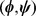 angles

Without loss of generality, we describe the sampling procedure for 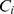 and 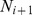 atoms at the 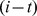-th growth step. 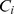 is generated first, followed by 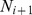. Denote the distance between 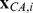 and 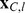 as 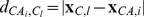, and the distance between 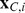 and 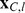 as 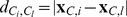. Since the bond angle 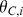 formed by the 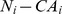 and 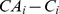 bonds is fixed, and the bond length 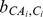 is also fixed, 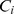 will be located on a circle 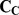 (Figure 3):
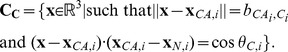
(1)Given a fixed 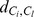, 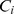 can be placed on two positions 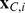 and 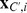 on circle 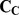 (Figure 3, 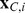 and 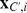 are labeled as 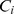 and 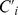, respectively.) As the probability for placing 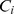 on either position is about equal based on our analysis, we randomly select one position to place atom 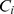.

In principle, sampling from the empirical distributions of 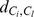 and mapping back to 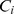 should encourage the growth of loops to connect to the terminal 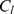 atom. Further analysis of the empirical distribution of 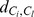 given 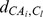 shows that 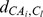 can be very informative for sampling 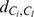 in some cases. This lead us to design the sampling of 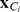 based on the conditional distribution of 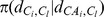. See below for details.

Generating atom 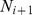 is similar to generating 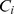, only 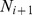 instead of 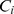 is placed on a circle 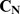:
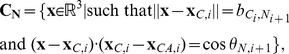
(2)where 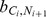 is the bond length between atom 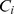 and atom 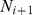, and the distance between 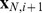 and 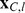 is 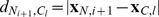. Similarly, atom 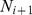 is placed by sampling 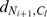 condition on 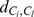 from the empirical conditional density 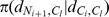. We repeat this process 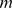 times to generate 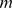 trial positions of 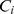, 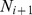, and 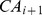.

#### Sampling 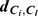 and 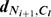 from conditional distributions

We sample 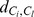 from the conditional distribution 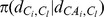 to obtain the location of 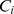 atom. We first construct the empirical joint distribution 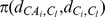 by collecting 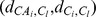 pairs over all loops in a loop database derived from the CulledPDB database (version 11118, at 30% identity, 2.0 Å resolution, and with 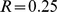) [56]. From the 6,521 protein structures in the CulledPDB, we remove 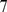 PDB structures which appear in our test data set. For the rest of 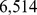 protein structures, loop regions were identified using the secondary structure information either directly from the PDB records or from classification provided by the DSSP software [57]. All random coil regions, including 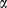-helices and 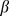-strands with length 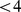 amino acids, are included in our database. In total, we have 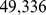 loop structures.

For each set of loops with the same residue separation 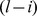, 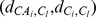 are Winsorised at 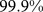 level [58]. Specifically, the extreme values above 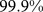 are replaced by the values at the 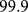 percentile. We then use a nonparametric two-dimensional Gaussian kernel density estimator to construct a smooth bivariate distribution 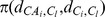 based on collected data. To estimate the probability density at a point 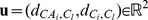, we use the observed 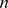 pairs of data from the database 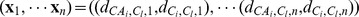 to derive the density function 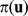, which takes the form of:
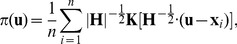
(3)where 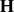 is the symmetric and positive definite bandwidth 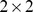 matrix, 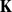 is a bivariate gaussian kernel function:
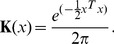
(4)


To construct the bandwidth matrix 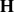, we calculate the standard deviation 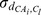 of the 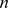 pairs of 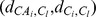. The corresponding entry 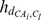 in the bandwidth matrix 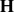 is set as 
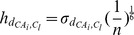
. Similarly, 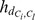 is set as 
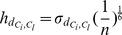
. The bandwidth matrix 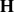 is then assembled as [59]:
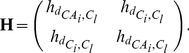
(5)We partition the domain of 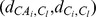 into a grid with 32 grid points in each direction. 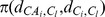 are estimated at the grid points, and interpolated by a bilinear function elsewhere. Conditional distribution 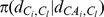 is constructed from the joint distribution 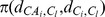 when 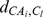 is fixed. 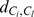 is sampled from 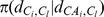. We follow the same procedure to construct 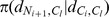, which is used to sample 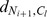.

#### Backbone dihedral angle distributions from the loop database

Although the empirical conditional distributions can efficiently guide chain growth to generate properly connected loop conformations, the dihedral angles of the loops are often not energetically favorable. As a result, conditional distributions described above alone are not sufficient in generating near native loop conformations.

The problem can be alleviated by an additional step of selecting a subset of 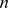 loops with low-energy dihedral angles from generated samples. We use empirical distributions of the loop dihedral angles obtained from the loop database. Specifically, for the 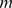 sampled positions of the current residue 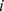 of type 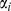 with dihedral angles 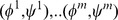, we select 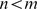 samples following an empirically derived backbone dihedral angle distribution 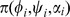. Here 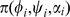 is derived from the same protein loop structure database for conditional distance distributions and constructed by counting the frequencies of 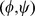 pairs for each residue type.

#### Determining the number of trial states at each growth step for backbone torsion angles

It is important to determine the appropriate size of trial states 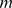 and 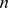 for generating backbone conformations, as small 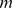 and 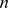 values may lead to insufficient sampling, resulting in inaccurate loop conformations. On the other hand, very large 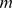 and 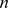 values will require significantly more computational time, without significant gain in accuracy.

We use a data set, denoted as *tuning-set* to determine the optimal values of parameters 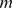 and 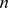 for sampling backbone conformations. Part of this data set comes from that of Soto *et al* [42]. The rest are randomly selected from pre-compiled CulledPDB (with 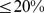 sequence identity, 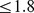 Å resolution, and 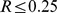). It contains a total of 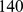 loops, with 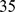 loops of length 6, 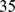 of length 8, 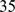 of length 10, and 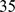 of length 12.

**Figure 4:**
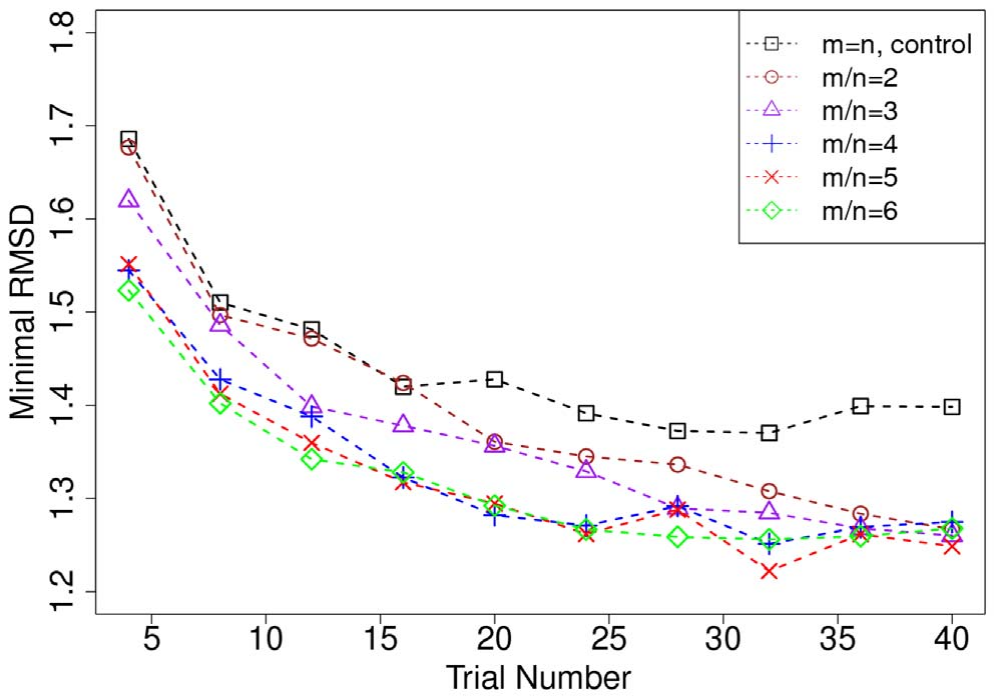
Mean of minimum backbone RMSD values for 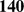 protein loops. We generated 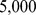 samples for each loop. The mean value of the minimum RMSD of the 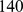 loops (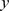-axis) is plotted against the size of trial samples 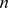 (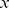-axis) for different choices of 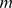. For control, results obtained without sampling torsion angles (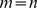, control) are also plotted. The backbone (N, 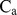, C and O atoms) RMSD in this paper is calculated by fixing the rest of the protein body.

The optimal values of 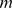 and 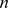 are determined as 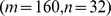 according to the test result on tuning-set (Figure 4).

#### Placement of backbone atoms

From the 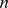 sampled dihedral angle pairs 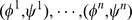, we can calculate the coordinates of atom 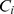 and 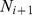 for all of the 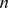 trials. 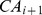 atoms are sampled by generating random 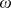 dihedral angles from a normal distribution with mean 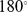 and standard deviation of 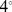. Calculating the coordinates of backbone 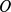 atoms using standard bond length and angle values is straightforward.

The coordinates of backbone atoms of the 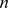 samples at this particular growth step can be denoted as 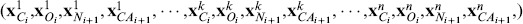. For simplicity, we denote the coordinates of the four atoms at residue 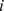 as 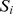 and the 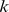-th sample as 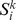. We sample one of them using an energy criterion. The probability for 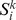 is defined by
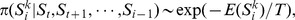
where 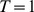 is the effective temperature, and 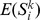 is the interaction energy of the four atoms defined by 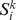 with the remaining part of the protein, including those loop atoms sampled in previous steps. The energy function 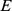 is an atomic distance-dependent empirical potential function constructed from the loop database, which is effective in detecting steric clashes and efficient to compute. Fragments with steric clashes are rarely drawn because of their high energy values. In summary, the coordinates of the four backbone atoms, 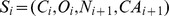, is drawn from the following joint distribution at this step:
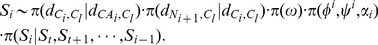
(6)Altogether, (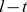) backbone dihedral angle combinations need to be sampled. When the growing end is three residues away from the 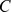-terminal anchor atom of the loop, 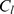, we apply the CSJD analytical closure method to generate coordinates of the remaining backbone atoms [12]. Small fluctuations of bond lengths, angles, and 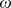 dihedral angles are introduced to the analytical closure method to increase the success rate of loop closure.

### Improving computational efficiency

To reduce computational cost of calculating atom-atom distances in energy evaluation, we use a procedure, REsidue-residue Distance Cutoff and ELLipsoid criterion (Redcell) to reduce computational time.

#### Residue-residue distance cutoff

The residue-residue distance cutoff 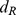 is used to exclude residues far from the loop energy calculation. Instead of a universal cutoff value, such as the 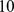 Å 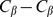 distance used in reference [51], we use a residue-dependent distance cutoff value. The residue-residue distance cutoff 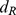 is assigned to be 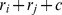, where 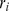 and 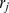 are the effective radii of residue 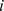 and 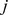, respectively. For one residue type, effective radii is the distance between residue geometrical center and the heavy atom which is farthest away from the residue geometrical center. 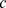 is a constant set to 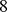 Å. For a residue 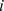 in the loop region and residue 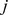 in the non-loop region, we calculate the residue-residue distance 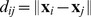, where 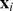 and 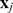 are the geometric centers of residue 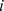 and 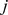, respectively. If 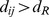, all of the atoms in residue 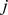 are excluded from energy calculation. This residue-dependent cutoff is more accurate and ensures close residues are included.

#### Ellipsoid criterion

**Figure 5:**
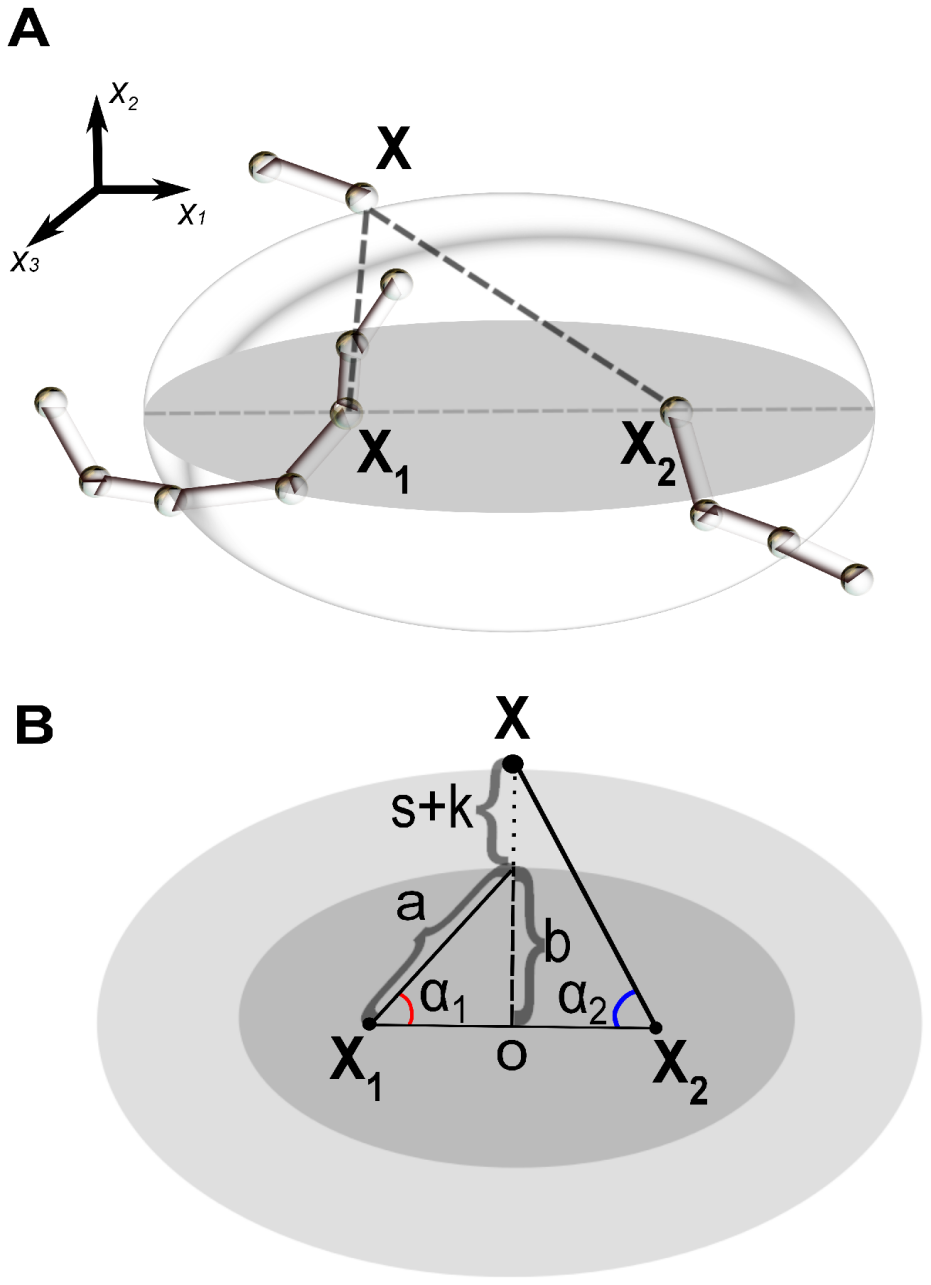
Schematic illustration of ellipsoid criterion. (A) Three dimensional view of a point 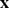 locating on the ellipsoid constructed from the total loop length 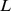 and the two foci 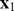 and 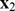. (B) Two dimensional view along through the 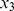-axis of the ellipsoid, with 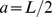 and 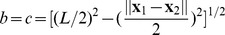 (dark gray). 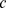 is along 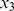-axis, not shown. The maximum side-chain length is denoted as 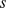 and the distance cut-off of interaction is 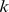. The enlarged ellipsoid, which has updated 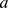 and 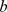, is also shown (light gray).

The basic idea of ellipsoid criterion is to construct a symmetric ellipsoid such that all atoms that need to be considered for energy calculation during loop sampling are enclosed in the ellipsoid. Atoms that are outside of the ellipsoid can then be safely excluded. The starting and ending residues of a loop naturally serve as the two focal points of the ellipsoid. Intuitively, all backbone atoms of a loop must be within an ellipsoid. Formally, we define a set of points 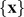, the sum of whose distances to the two foci is less than 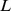, defined as the sum of the backbone bond lengths 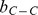 of the loop of length 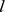:
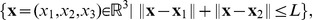


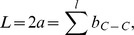
where 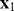 and 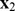 are the two focal points of the ellipsoid. The symmetric ellipsoid (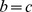) can be written as:
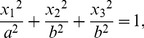
(7)where 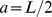 and 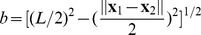 correspond to the semi-major axis and semi-minor axis of the symmetric ellipsoid, respectively. To incorporate the effects of side chain atoms, we enlarge the ellipsoid by the amount of the maximum side-chain length 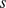. Furthermore, we assume that any atom can interact with a loop atom if it is within a distance cut-off of 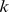. As a result, the overall enlargement of the ellipsoid is 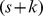. The final definition of the enlarged ellipsoid for detecting possible atom-atom interactions is given by Eqn (7), with
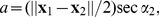
(8)and
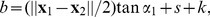
(9)where 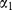 is determined by the equation 
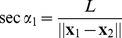
, and 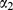 by 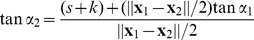 (see Figure 5B).

For any atom in the protein, if the sum of its distances to the two foci points is greater than 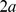, this atom is permanently excluded from energy calculations. The computational cost to enforce this criterion depends only on the loop length and is independent of the size the protein, once the rest of the residues have been examined using the ellipsoid criterion. This improves our computing efficiency significantly, especially for large proteins. This criterion also helps to prune chain growth by terminating a growth attempt if the placed atoms are outside the ellipsoid.

### Side-chain modeling and steric clash removal

Side chains are built upon completion of backbone sampling of a loop. For the 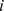-th residue of type 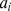, we denote the degrees of freedom (DOFs) for its side chain as 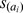. DOFs of side chain residues depend on the residue types, *e.g.* Arg has four dihedral angles (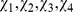), with (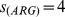). Val only has one dihedral angle (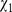), with (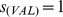). Each DOFs is discretized into bins of 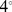, and only bins with non-zero entries for all loop residues in the loop database are retained.

We sample 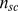 trial states of side chains from the empirical distribution 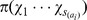 obtained from the loop database. One of 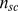 trials is then chosen according to the probability calculated by the empirical potential. Denote the side chain fragment for the 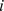-th residue as 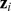, we select 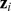 following the probability distribution:
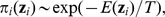
where 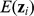 is the interaction energy of the newly added side chain fragment 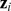 with the remaining part of the protein, and 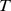 is the effective temperature.

When there are steric clashes between side chains, we rotate the side-chain atoms along the 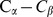 axis for all residue types except Pro. For Pro, we use the 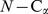 axis for rotation. We consider two atoms to be in steric clash if the ratio of their distance to the sum of their van der Waals radii is less than 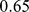 [13].

### Potential function

To evaluate the energy of loops, we develop a simple atom-based distance-dependent empirical potential function, following well-established practices [46], [52], [60]–[66]. Empirical energy functions developed from databases have been shown to be very effective in protein structure prediction, decoy discrimination, and protein-ligand interactions [54], [63], [64], [67]–[71]. As our interest is modeling the loop regions, the atomic distance-dependent empirical potential is built from loop structures collected in the PDB [72].

Instead of using detailed 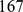 atom types associated with the 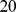 amino acids, we group all heavy atoms into 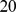 groups, similar to the approach used in Rosetta [50]. The 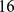 side-chain atom types comprise six carbon types, six nitrogen types, three oxygen types, and one sulfur type. The 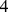 backbone types are N, 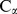, C, and O. This simplified scheme helps to alleviate the problem of sparsity of observed data for certain parameter values. For an atom 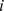 in the loop region of atom type 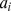 and an atom 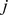 of atom type 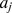, regardless whether 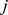 is in the loop region, the distance-dependent interaction energy 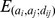 is calculated as :
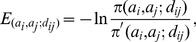
(10)where 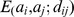 denotes the interaction energy between a specific atom pair 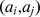 at distance 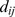, 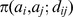 and 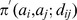 are the observed probability of this distance-dependent interaction from the loop database and the expected probability from a random model, respectively.

The observed probability 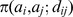 is calculated as: 
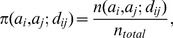
(11)where 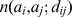 is the observed count of 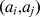 pairs found in the loop structures with the distance 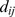 falling in the predefined bins. We use a total of 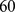 bins for 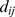, ranging from 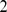 Å to 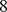 Å, with the bin width set to 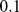 Å. 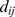 ranging from 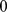 Å to 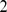 Å is treated as one bin. Here 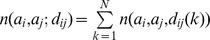, where 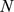 is the number of loops in our loop database, 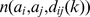 is the observed number of 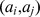 pairs at the distance of 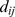 in the 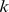-th loop. 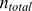 is the observed total number of all atom pairs in the loop database regardless of the atom types and distance, namely, 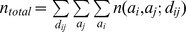.

The expected random distance-dependent probability of this pair 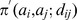 is calculated based on sampled loop conformations, called decoys. It is calculated as: 
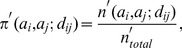
(12)where 
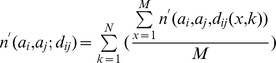
 is the expected number of (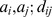) pairs averaged over all decoy loop conformations of all target loops in the loop database. Here 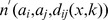 is the number of 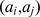 pairs at distance 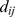 in the 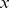-th generated loop conformations for the 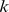-th loop. 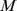 is the number of decoys generated for a loop, which is set to 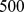. 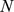 is the number of loops in our loop database. 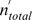 is the total number of all atom pairs in the reference state, 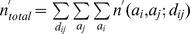.

### Tool availability

We have made the source code of DiSGro available for download. The URL is at: tanto.bioengr.uic.edu/DiSGro/.

## Supporting Information

Text S1 **Results of modeled loops on Test Set 2–5, calculated using DiSGro.** Table 1–3 are tables for Test Set 2. Table 4–12 are tables for Test Set 3. Table 13–18 are tables for Test Set 4. Table 19–22 are tables for Test Set 5.
(PDF)
