# Supplementary material for: Fast Protein Loop Sampling and Structure Prediction Using Distance-Guided Sequential Chain-Growth Monte Carlo Method
Source: PLoS Comput Biol. 2014 Apr 24;10(4):e1003539. doi: 10.1371/journal.pcbi.1003539 (PMC3998890; doi:10.1371/journal.pcbi.1003539)
Supplement: Text S1 — Results of modeled loops on Test Set 2–5, calculated using DiSGro. Table 1–3 are tables for Test Set 2. Table 4–12 are tables for Test Set 3. Table 13–18 are tables for Test Set 4. Table 19–22 are tables for Test Set 5. (PDF) [file pcbi.1003539.s001.pdf]

Text S1

Table 1: **The average minimal RMSD (R_{min}) of 8-residue loops on Test Set 2, calculated using DiSGro.**

PDB	Start	End	Sequence	R_{min}
1a1h	107	114	CPVESCDR	1.38
1a3c	92	99	IPVDITDQ	0.88
1a62	70	77	SSYLAGPD	0.65
1a62	102	109	RPPKEGER	0.68
1aac	49	56	EAMPHNVH	0.3
1aba	7	14	YDSNIHKC	0.62
1ads	274	281	KVFDFELS	0.77
1amm	69	76	MGFNDSIR	0.88
1amm	158	165	GAMNAKVG	0.41
1arb	136	143	RRDQNYPG	1.11
1arb	212	219	GPSSCSAT	0.69
1arb	249	256	DPASTGAQ	0.95
1aru	234	241	LSPFPGEF	0.41
1brt	205	212	WYTDFRAD	0.49
1btk	67	74	VVPEKNPP	0.8
1cex	73	80	VGGAYRAT	1.84
1cvl	148	155	TLVSSSHN	0.84
1cvl	229	236	TSTGTLDV	0.39
1dad	176	183	DVTPPGKR	0.55
1dim	246	253	RNSGLRRS	0.7
1ezm	92	99	GTSPLTHK	0.41
1hfc	119	126	ENYTPDLP	1.36
1kpf	105	112	GQSVYHVH	0.71
1mrp	68	75	AGLLAPIS	0.78
1msi	26	33	VVTPVGIP	1.13
1nfp	118	125	NVDIANVR	1.05
1nif	221	228	NGAVGALT	0.87
1nif	279	286	ETWLIPGG	0.8
1nls	97	104	TGLYKETN	0.73
1nox	99	106	VIHPGVQG	1.12
1opd	8	15	ITAPNGLH	0.54
1plc	6	13	GADDGSLA	1.24
1plc	32	39	NAGFPHNI	1.16
1ppn	101	108	GPYAAKTD	0.85
1ppn	191	198	RGTGNSYG	0.58

1ra9	51	58	GRPLPGRK	0.77
1rhs	235	242	KKVDLTKP	0.47
1rro	18	25	CQDPDTFE	0.59
1vwj	45	52	SAVGNAES	1.28
1wer	824	831	SKQSCELS	0.37
1wer	916	923	NIISDSPS	0.55
2arc	28	35	ANGYLDFF	0.49
2ayh	124	131	TNGVGGHE	0.65
2ayh	194	201	GSYNGANP	0.66
2ctc	53	60	STGGSNRP	1.45
3seb	40	47	SIDQFLYF	0.5
5p21	144	151	TSAKTRQG	0.59
5ptp	22	29	CGANTVPY	0.78
5ptp	172	179	YPGQITSN	1.36
7rsa	64	71	ACKNGQTN	0.85
Mean				**0.80**

Table 2: **The average minimal RMSD ($R_{\min}$) of 11-residue loops on Test Set 2, calculated using DISGRO.**

PDB	Start	End	Sequence	$R_{\min}$
153l	154	164	VRSYARMDIGT	0.94
1a2p	74	84	INYTSGFRNSD	1.47
1a2y	91	101	FWSTPRTFGGG	1.54
1ads	290	300	SYNRNWRVCAL	0.66
1akz	130	140	AHQANSHKERG	1.29
1aru	289	299	IPSAVSNNAAP	1.56
1awq	100	110	ANAGPNTNGSQ	1.41
1cvl	254	264	RASGQNDGLVS	1.16
1dad	42	52	GSEKTPEGLRN	1.52
1ixh	120	130	NPGLKLPSQNI	1.09
1mla	7	17	PGQGSQTVGML	0.96
1nls	216	226	NIDSSIPSGST	1.28
1rcf	122	132	TDGYDFNDSKA	1.3
2eng	120	130	IPGGGVGIFDG	0.5
2pth	8	18	LANPGAEYAAT	1.28
3pte	91	101	LPGLLPDDRIT	1.18
5pti	7	17	EPPYTGPCKAR	1.09
Mean				**1.19**

Table 3: **The average minimal RMSD ($R_{\min}$) of 12-residue loops on Test Set 2, calculated using DISGRO.**

PDB	Start	End	Sequence	$R_{\min}$
153l	98	109	KRSHKPQGTWNG	1.15
1akz	100	111	IEDFVHPGHGDL	0.86
1arb	74	85	NYQNSTCRAPNT	1.2
1bkf	9	20	PGDGRTFPKRGQ	1.14
1cex	24	35	RGSTETGNLGTL	0.91
1dim	212	223	TDGITWSLPSGY	0.87
1ixh	160	171	VGTGSTVKWPIG	1.86
1luc	158	169	LNPSAYTQGGAP	1.99
1xyz	298	309	NPLIYDSNYNPK	1.15
2ayh	21	32	ADGYSNGGVFNC	1.62
Mean				**1.28**

Table 4: **Results of 4-residue loops on Test Set 3, calculated using DISGRO.**

PDB	Start	End	Sequence	R_{min}	R_{ave}	R_{Emin}
1aaj	82	85	FTEA	0.08	0.44	0.39
1ads	99	102	LKLD	0.12	0.27	0.26
1cbs	21	24	VLGV	0.3	0.79	0.85
1fkf	42	45	RNKP	0.1	0.3	0.36
1frd	59	62	DQSD	0.18	0.98	0.24
1gpr	123	126	NVPS	0.43	1.11	0.7
1iab	100	103	FYHE	0.11	0.52	0.11
1mba	97	100	GFGV	0.09	0.33	0.49
1nfp	37	40	EDTS	0.64	1.56	0.93
1pbe	117	120	GATT	0.23	0.92	0.97
1pgs	226	229	LGAL	0.19	1.38	0.19
1plc	74	77	LSNK	0.1	0.45	0.26
1ppn	42	45	TGNL	0.13	0.5	0.33
1prn	66	69	GNAA	0.19	0.54	0.31
1rcf	111	114	QRGG	0.3	0.61	0.53
1tca	287	290	AGPK	0.14	0.88	0.24
1thw	194	197	PGSS	0.15	0.39	0.22
1tib	46	49	KADA	0.07	0.27	0.14
1tml	42	45	FAHH	0.66	1.19	1.08
1tys	131	134	SAWN	0.11	0.55	1.2
1xif	82	85	TGMK	0.11	0.28	0.31
1xnb	30	33	WSNT	0.02	0.08	0.06
2cmd	163	166	GKQP	0.24	1.11	0.57
2cy3	101	104	KDKK	0.08	0.33	0.37
2exo	161	164	DPTA	0.42	0.79	0.84
2sga	44	47	LGFN	0.17	0.79	0.17
2sil	220	223	LPSG	0.84	1.17	1.2
2tgi	72	75	ASAS	0.11	0.4	0.45
3cyr	69	72	HAKG	0.31	0.71	0.91
4enl	335	338	EKKA	0.05	0.27	0.16
4gcr	116	119	FHLT	0.31	0.84	0.76
5fd1	81	84	ITEK	0.17	0.7	0.42
5p21	75	78	GEGF	0.03	0.75	0.2
7rsa	47	50	VHES	0.12	0.54	0.21
8abp	55	58	ASGA	0.1	0.32	0.22
Mean				**0.21**	**0.66**	**0.48**

R_{min}, R_{ave} and R_{Emin} denote the average minimum backbone RMSD, the average ensemble RMSD and the average RMSD of the lowest energy conformations of the 1,000 loop ensemble with the same length, respectively.

Table 5: **Results of 5-residue loops on Test Set 3, calculated using DiSGro.**

PDB	Start	End	Sequence	R_{min}	R_{ave}	R_{Emin}
1ads	274	278	KVFDF	0.27	1.16	1.64
1alc	110	114	LCTEK	0.48	2.15	0.66
1art	346	350	ANRDF	0.28	1.42	1.12
1byb	223	227	RAGHP	0.17	0.73	0.55
1csh	98	102	TGQIP	0.36	1.58	1.91
1cus	31	35	CADVI	0.27	0.96	0.3
1dts	62	66	SSLQL	0.36	1.2	1.35
1eco	72	76	ELPNI	0.55	1.72	2.11
1ede	15	19	LDQYP	0.38	1.95	0.43
1fkf	10	14	GDGRT	0.16	1.33	0.38
1frd	83	87	PRSNC	0.16	0.91	0.7
1gof	430	434	FASNG	0.22	0.82	0.44
1gpr	54	58	SPVRG	0.27	0.78	0.91
1hbq	158	162	ELCLA	0.11	0.55	0.56
1nar	56	60	ESWDV	0.19	0.95	0.68
1onc	59	63	TTSEF	0.14	0.71	0.92
1pbe	194	198	PVSHE	0.1	0.7	0.36
1pgs	36	40	DVTTV	0.13	0.84	0.38
1phf	277	281	RPERI	0.33	2.05	0.92
1poa	108	112	NDYNI	0.08	0.61	0.62
1prn	187	191	GIVDN	0.16	0.8	0.3
1sbp	181	185	VERGI	0.3	1.77	0.57
1tib	253	257	PDIPA	0.07	0.51	0.31
1tml	147	151	GSSQA	0.13	0.48	0.46
1ukz	94	98	KANKH	0.12	0.68	0.46
1ycc	74	78	YIPGT	0.34	1.06	0.91
2cmd	188	192	QVPGV	0.49	1.31	1.22
2cpl	115	119	CTAKT	0.29	1	0.81
2end	93	97	ISDIP	0.22	1.52	1.49
2fox	25	29	ESGKD	0.25	1	1.11
2hbg	37	41	AHPQM	0.17	1.16	0.17
3tgl	207	211	HLPPA	0.86	1.52	1.16
7rsa	75	79	SYSTM	0.15	0.81	0.75
8abp	300	304	KKGLG	0.04	0.86	1.91
Mean				**0.25**	**1.11**	**0.84**

R_{min}, R_{ave} and R_{Emin} denote the average minimum backbone RMSD, the average ensemble RMSD and the average RMSD of the lowest energy conformations of the 1,000 loop ensemble with the same length, respectively.

Table 6: **Results of 6-residue loops on Test Set 3, calculated using DISGRO.**

PDB	Start	End	Sequence	R_{min}	R_{ave}	R_{Emin}
1ads	149	154	DEGLVK	0.17	0.67	0.46
1arp	282	287	VLGFDR	0.41	2.73	1.24
1cbs	66	71	KVGEEF	0.31	0.9	1.42
1dts	146	151	GVKLGC	0.61	2.35	3.16
1ede	180	185	VTPSDL	0.97	2.28	3.2
1fnd	204	209	VPTSSS	0.36	0.88	0.41
1gca	100	105	DSYDKA	0.26	0.95	0.4
1gpr	83	88	HFGIDT	0.63	1.5	0.93
1hbq	15	20	FDKARF	0.52	1.89	0.7
1lst	109	114	LKGKHV	0.32	1.02	0.51
1onc	12	17	TNTRDV	0.28	1.72	0.28
1phf	350	355	FGHGSH	0.38	1.21	1.63
1php	217	222	IGGGLA	0.21	0.73	0.38
1ppn	144	149	YRGGIF	0.44	3.43	0.7
1prn	57	62	DGDAFA	0.33	2.65	2.61
1scs	19	24	DPSYPH	0.4	1.32	0.96
1tca	94	99	SGNNKL	0.33	1.48	0.7
1tib	176	181	VGNRAF	0.52	1.34	0.53
1tys	66	71	DTNIAY	0.23	0.9	0.23
1xif	357	362	FEEFDV	0.85	3.78	2.9
1ycc	85	90	LKKEKD	0.29	1.76	0.29
2ayh	160	165	VDGVLK	0.3	2.83	0.67
2cpl	122	127	LDGKHV	0.19	0.91	0.4
3cyr	25	30	HEKVEC	0.27	0.99	0.96
2mnr	308	313	AATPTA	0.39	0.91	0.47
2ran	40	45	LTARSN	0.26	1.37	0.47
2sil	176	181	GGVGSG	0.24	1.14	0.33
3chy	12	17	DDFSTM	0.42	2.19	3.71
3grs	336	341	ALLTPV	0.81	2.91	2.14
3hsc	227	232	HLGGED	0.31	1.39	0.37
3tgl	82	87	SSSIRN	0.63	2.46	1.74
5fd1	87	92	PLPDAE	0.86	2.58	0.99
5fx2	41	46	VEAGGL	0.95	2.02	3.65
5p21	104	109	KDSDDV	0.26	1.31	1.06
7rsa	14	19	DSSTSA	0.64	2.06	1.51
8abp	65	70	TPDPKL	0.47	2.05	1.76
Mean				**0.44**	**1.74**	**1.22**

R_{min}, R_{ave} and R_{Emin} denote the average minimum backbone RMSD, the average ensemble RMSD and the average RMSD of the lowest energy conformations of the 1,000 loop ensemble with the same length, respectively.

Table 7: **Results of 7-residue loops on Test Set 3, calculated using DISGRO.**

PDB	Start	End	Sequence	$R_{\min}$	R_{ave}	R_{Emin}
1amp	225	231	YACSDHA	0.54	2.12	1.13
1arb	249	255	DPASTGA	0.36	2.47	3.46
1art	356	362	QNGMFSF	0.49	1.31	0.56
1byb	198	204	WEFPRIG	0.97	1.67	1.16
1cbs	45	51	QEGDTFY	0.9	4.62	1.48
1cyo	24	30	ILHYKVY	0.37	1.44	0.46
1dts	76	82	FAEPTSP	0.67	1.5	1.25
1ede	121	127	VVQDWGG	0.6	2.04	1.89
1gca	196	202	LSGPNAN	0.46	2.65	0.67
1gpr	35	41	SGKMMGD	0.54	2.72	1.62
1hbq	138	144	ARDPSGF	0.49	1.54	1.54
1hfc	151	157	KVSEGQA	0.3	0.99	0.64
1iab	142	148	QYYSIMH	0.38	1.44	0.44
1lif	64	70	FKLGVEF	0.55	2.91	1.65
1lst	117	123	LQGSTQE	0.22	2.55	0.3
1mbd	17	23	VEADVAG	0.43	1.67	1.02
1phf	26	32	FDMYNPS	0.53	1.43	1.03
1php	135	141	AELADLY	0.34	1.08	0.65
1plc	87	93	HQGAGMV	0.4	1.39	0.52
1pmy	25	31	LKPGDSI	0.42	1.3	0.42
1ppn	166	172	YGPNYIL	0.75	2.54	1
1ptf	65	71	VDGADEA	0.5	3.15	0.61
1sbp	169	175	LDSGARG	0.39	4.24	1.1
1thw	158	164	CCTTGKC	0.53	2.12	0.91
1tml	20	26	NPNDPRT	0.59	2.69	1.45
1ycc	57	63	VLWDENN	0.78	2.07	0.89
2alp	130	136	AAVGAAV	0.3	1.87	0.3
2ayh	54	60	SAYNKFD	0.93	3.31	1.95
2cba	61	67	NNGHAFN	0.54	1.81	1.49
2ctc	53	59	STGGSNR	0.88	2.9	1.79
2mnr	270	276	DAMKIGG	0.39	1.34	0.39
3grs	26	32	IGGGSGG	0.58	1.62	0.78
3tgl	159	165	QREEGLS	0.6	2.99	1.9
4fgf	58	64	EERGVVS	1.03	3.6	1.03
4gcr	150	156	EYRRYLD	0.34	2.04	0.34
5fx2	27	33	ADAGYEV	0.4	3.85	1.05
5p21	83	89	AINNTKS	0.4	1.4	0.4
7rsa	20	26	ASSSNYC	0.94	2.47	1.91
Mean				**0.55**	**2.23**	**1.08**

$R_{\min}$, R_{ave} and R_{Emin} denote the average minimum backbone RMSD, the average ensemble RMSD and the average RMSD of the lowest energy conformations of the 1,000 loop ensemble with the same length, respectively.

Table 8: **Results of 8-residue loops on Test Set 3, calculated using DISGRO.**

PDB	Start	End	Sequence	R_{min}	R_{ave}	R_{Emin}
135l	84	91	LSSDITAS	0.53	2.37	1.98
1alc	34	41	SGYDTQAI	0.5	5.02	0.53
1art	88	95	FGKGSALI	0.98	3.57	3.8
1btl	50	57	DLNSGKIL	0.75	2.19	1.2
1cbs	55	62	STTVRTTE	0.58	4.67	5.85
1ddt	127	134	FGDGASRV	0.89	2.41	2.5
1fnd	262	269	LKKDNTYV	0.3	2	1.49
1gky	72	79	QFSGNYYG	0.7	5.22	0.72
1hfc	142	149	SNVTPLTF	0.67	1.38	1.32
1iab	48	55	RTTESDYV	1.17	2.89	1.45
1nar	192	199	FSNQQKPV	0.61	1.67	1.03
1oyc	80	87	GGYDNAPG	0.77	2.09	1.66
1phf	85	92	CPFIPREA	1.11	2.32	1.95
1poa	71	78	CSQGTLTC	1.03	2.42	2.05
1prn	150	157	DPDQTVDS	0.59	2.81	1.75
1sbp	107	114	KQIHDWND	0.49	3.27	0.5
1thw	18	25	SKGDAALD	0.57	2.17	1.34
1tml	187	194	NTSNYRWT	0.53	1.58	0.73
1tys	83	90	WADENGDL	0.99	5.11	2.77
1xnb	99	106	KSDGGTYD	0.54	1.85	1.63
2ayh	123	130	YTNGVGGH	0.59	1.9	0.65
2cmd	270	277	LGKNGVEE	0.65	2.64	1.12
2ctc	89	96	DYGQDPSF	0.92	2.03	1.5
2dri	161	168	PADFDRIK	0.65	5.92	0.65
2exo	262	269	MQVTRCQG	0.42	1.53	0.89
2fox	88	95	YGWGDGKW	1.07	2.58	1.41
2ran	26	33	MKGLGTDE	1.43	3.08	2.92
2sga	32	43	TTGGSRCS	0.9	1.8	2.09
3grs	424	431	ANKEEKVV	1.82	5.77	2
4enl	24	31	TTEKGVFR	0.58	1.89	1.68
5p21	45	52	VIDGETCL	0.51	1.97	1.42
8dfr	65	72	RPLKDRIN	1.65	3.68	2.56
Mean				**0.80**	**2.87**	**1.72**

R_{min}, R_{ave} and R_{Emin} denote the average minimum backbone RMSD, the average ensemble RMSD and the average RMSD of the lowest energy conformations of the 1,000 loop ensemble with the same length, respectively.

Table 9: **Results of 9-residue loops on Test Set 3, calculated using DISGRO.**

PDB	Start	End	Sequence	R_{min}	R_{ave}	R_{Emin}
1aba	69	77	FAPDGSHIG	0.71	2.77	1.18
1amp	57	65	SASLPNASV	0.76	2.07	1.22
1arp	127	135	SNCPGSPRL	0.67	1.99	1.39
1btl	102	110	LVEYSPVTE	1.15	4.99	1.64
1byb	246	254	GFFKSNGTY	1.23	6.89	1.89
1csh	252	260	GSALSDPYL	0.91	3.57	1.5
1cyo	49	57	QAGGDATEN	0.73	2.93	0.87
1ede	257	265	GMKDKLLGP	1.07	5.69	1.44
1flp	41	49	AKFSGLFSG	0.67	4.17	3.31
1fnd	121	129	TNDAGETIK	0.71	4.32	5.86
1fus	31	39	DTAGSSTYP	1.3	4.44	3.22
1gca	9	17	IYKYDDNFM	0.91	2.57	2.22
1gky	6	14	ISGPSGTGK	0.52	2.59	0.76
1gpr	63	71	VFPTKHAIG	1.33	4.06	1.75
1lif	73	81	ITADDRKVK	1.21	2.72	2.08
1mrk	53	61	TNYADETIS	1.11	2.43	1.62
1noa	76	84	FLFDGTRWG	1.12	3.52	1.31
1npk	102	110	ASAPGSIRG	0.61	5.29	0.61
1onc	70	78	VTSRPCKYK	1.57	6.65	2.01
1pgs	117	125	TETWLAKGR	0.59	1.98	1.39
1php	91	99	TNEAVGDEV	0.9	4.54	1.13
1ptf	10	18	AETGIHARP	0.89	3.77	2.07
1tib	69	77	LDNTNKLIV	0.74	2.05	1.82
1xif	59	67	IPFGSSDSE	0.78	5.01	1.54
1xnb	116	124	PSIDGDRTT	0.96	2.58	1.89
2alp	139	159	SGRTTGYQC	0.79	2.33	1.35
2ayh	41	49	FTNDGKLKL	1.05	3.47	1.51
2cmd	81	89	RKPGMDRSD	0.85	4.81	2.93
2cpl	24	32	LFADKVPKT	0.74	3.34	0.97
2dri	130	138	QGIAGTSAA	0.79	2.69	1.17
2fox	5	13	YWSGTGNTE	0.85	3.35	3
2hbg	18	26	AGADNGAGV	1.2	4.44	3.11
2sil	183	191	QLNDGKLVF	0.91	2.63	0.91
3chy	57	65	DWNMPNMDG	0.84	3.01	0.84
3tgl	56	64	STLIYDTNA	1.64	4.08	2.24
4gcr	94	102	ERDDFRGQM	0.96	3.75	1.68
5fx2	8	16	YGSTTGNTE	1.06	3.19	1.81
Mean				**0.94**	**3.64**	**1.82**

R_{min}, R_{ave} and R_{Emin} denote the average minimum backbone RMSD, the average ensemble RMSD and the average RMSD of the lowest energy conformations of the 1,000 loop ensemble with the same length, respectively.

Table 10: **Results of 10-residue loops on Test Set 3, calculated using DISGRO.**

PDB	Start	End	Sequence	R_{min}	R_{ave}	R_{Emin}
135l	18	27	DNYRGYSLGN	1.53	4.04	2.53
1ads	170	179	LNKPGLKYKP	1.09	4.24	2.13
1amp	181	190	TNYKGSAQDV	1.01	2.81	1.89
1arp	37	46	FYQGSKCESP	1.02	5	1.34
1btl	170	179	NEAIPNDERD	0.72	2.62	2.27
1ede	164	173	FVTQPADGFT	2.09	7.23	2.3
1fkf	63	72	VAQMSVGQRA	0.48	2.97	0.48
1fnd	185	194	FFEKHDDYKF	1.69	3.32	3.73
1gpr	133	142	FTNLAEGETV	1.13	3.52	2.6
1hfc	201	210	ERWTNNFREY	1.07	4.8	1.87
1knt	35	44	YGGCGGNENK	1.27	4.17	2.19
1lst	10	19	TDTTYAPFSS	1.38	3.58	1.67
1mbd	40	49	LEKFDRFKHL	1.19	3.12	3.9
1nfp	53	62	DKSYNDETKL	1.6	7.43	8.15
1onc	48	57	CKGIIASKNV	1.01	6.77	1.41
1pbe	157	166	GCDGFHGISR	1.52	4.28	4.02
1pgs	68	77	KNKTTGEWYE	0.99	2.69	2.23
1ppn	190	199	KRGTGNSYGV	1.48	5.08	2.5
1prn	118	127	NSKYDASGAL	1.26	4.88	5.83
1sbp	124	133	TPNPKSSGGA	0.6	1.56	0.65
1scs	65	74	VSYPNADSAT	0.95	2.48	1.62
1tib	237	246	KIEGIDATGG	0.67	3.2	2.31
1ukz	21	30	VLGGPGAGKG	1.16	4.27	2.72
2alp	90	105	RVFPGNDRAW	1.21	3.36	2.23
2ayh	80	89	KPAKNTGIVS	0.98	2.66	1.74
2cmd	57	66	GFSGEDATPA	0.54	3.61	0.54
2ctc	66	75	LGIHSREWIT	1.23	2.57	2.01
2mnr	91	100	FCLAGYTGLI	1.32	4	1.57
2sil	197	206	RTKNITTVLN	1.44	3.56	1.88
2sn3	30	39	KAKNQGGSYG	1.06	4.51	1.72
3hsc	28	37	IIANDQGNRT	1.11	3.24	2.95
3tgl	257	266	HLSYFGINTG	0.64	2.64	0.64
4fgf	42	51	GVREKSDPHI	0.69	2.95	1.3
5fx2	59	68	TWGDDSIELQ	1.7	4.58	2.63
5p21	8	17	VVGAGGVGKS	1.17	2.9	1.98
5pti	23	32	YNAKAGLCQT	0.77	2.71	2.36
7rsa	110	119	CEGNPYVPVH	1.83	9.08	2.28
Mean				**1.15**	**3.96**	**2.33**

R_{min}, R_{ave} and R_{Emin} denote the average minimum backbone RMSD, the average ensemble RMSD and the average RMSD of the lowest energy conformations of the 1,000 loop ensemble with the same length, respectively.

Table 11: **Results of 11-residue loops on Test Set 3, calculated using DISGRO.**

PDB	Start	End	Sequence	R_{min}	R_{ave}	R_{Emin}
1aaj	91	101	HCTPHPFMRGK	0.99	2.25	1.67
1acf	58	68	GGFDLAGVHYV	1.24	10.51	1.89
1amp	32	42	FTNRFYTTTSG	1.58	6.7	2.52
1arb	212	222	GPSSCSATGTN	2.18	9.1	2.18
1art	189	199	HGCCHNPTGID	1.47	2.72	2.42
1byb	22	32	VVNVDNVFEDP	2.26	4.72	3.1
1cid	121	131	VMGPTSPKMRL	1.09	2.76	2.41
1esl	144	154	CDPGFSGLKCE	1.58	5.86	4.29
1ezm	24	34	LIVNDRCEMDD	1.16	4.71	2.09
1iab	79	89	LQANGCVYHGT	1.71	5.53	1.81
1knb	521	531	YLNGDKTKPVT	1.5	3.67	2.29
1mrk	92	102	AKYVFKDAMRK	1.75	3.85	2.66
1noa	97	107	LSDAAGNGPEG	1.45	4.45	5.43
1phf	100	110	PTSMDPPEQRQ	1.56	3.77	2.73
1php	269	279	ADRFANDANTK	1.5	2.89	2.94
1pii	282	292	IFVATSPRCVN	1.47	5.97	2.03
1plc	5	15	LGADDGSLAFV	1.36	6.71	1.89
1tca	68	78	PPPFMLNDTQV	2.16	6.19	5.16
1trb	87	97	VDLQNRPFRLN	1.38	5.79	2.15
1xnb	41	51	GWTTGSPFRTI	1.15	3.61	1.4
2exo	170	180	DYNVEGINAKS	1.21	4.78	2
2hbg	45	55	FGFSGASDPGV	0.97	4.99	1.25
2pia	74	84	KRDSNGRGGSI	1.47	3.71	2.74
2pgd	222	232	WNKTELDSFLI	1.19	4.85	6.11
2rhe	38	48	QQVPGKAPKLL	1.3	7.01	8.26
3grs	267	277	TAVPGRLPVMT	1.37	7.36	8.88
3hsc	56	66	KNQVAMNPTNT	1.13	3.49	3.84
3tgl	98	108	SYPPVSGTKVH	1.31	3.66	2.78
4enl	206	216	GNVGDEGGVAP	1.35	5.72	1.45
4i1b	123	133	STSQAENMPVF	0.89	3.29	1.05
5p21	144	154	TSAKTRQGVED	0.83	3.39	2.21
6taa	150	160	CFIQNYEDQTQ	1.25	6.13	2.12
8dfr	39	49	STSHVEGKQNA	1.04	3.7	2.46
Mean				**1.39**	**4.96**	**2.98**

R_{min}, R_{ave} and R_{Emin} denote the average minimum backbone RMSD, the average ensemble RMSD and the average RMSD of the lowest energy conformations of the 1,000 loop ensemble with the same length, respectively.

Table 12: **Results of 12-residue loops on Test Set 3, calculated using DISGRO.**

PDB	Start	End	Sequence	R_{min}	R_{ave}	R_{Emin}
154l	153	164	NVRSYARMDIGT	1.43	4.47	3.23
1arp	201	212	LDSTPQVFDTQF	1.23	4.38	1.48
1ctm	9	20	YENPREATGRIV	1.62	5.44	1.62
1cyo	12	23	IQKHNNSKSTWL	1.04	4.25	2.93
1dts	41	52	SGSEKTPEGLRN	1.9	11.47	6.24
1eco	35	46	MAKFTQFAGKDL	1.58	3.63	2.45
1ede	150	161	CLMTDPVTQPAF	1.52	3.3	1.94
1ezm	122	133	FGDGATMFYPLV	1.17	3.25	1.29
1hfc	165	176	RGDHRDNSPFDG	1.96	7.61	2.27
1msc	9	20	LVDNGGTGDVTV	1.77	6.42	7.66
1onc	23	34	MSTNLFHCKDKN	2.41	5.37	3.77
1pbe	129	140	LHDLQGERPYVT	1.33	6.05	2.12
1pmy	77	88	KCAPHYMMGMVA	1.42	3.15	2.56
1prn	15	26	VEDRGVGLEDTI	1.83	5.46	5.99
1rcf	88	99	TGDQIGYADNFQ	1.44	5.46	1.49
1rro	17	28	ECQDPDTFEPQK	1.17	4.5	1.17
1scs	199	210	IKSPDSHPADGI	0.9	2.98	2.46
1srp	311	322	SDVGGLKGNVSI	0.68	2.25	0.68
1tca	305	316	AVGKRTCSGIVT	1.65	11.17	8.24
1thg	127	138	WIYGGAFVYGSS	1.49	3.04	2.16
1thw	178	189	PDAFSYVLDKPT	1.17	6.67	1.27
1tib	99	110	EINDICSGCRGH	1.49	3.71	2.11
1tml	243	254	STTNTGDPMIDA	1.99	5.79	2.52
1xif	203	214	IERLERPELYGV	1.25	3.09	1.93
2cpl	145	156	FGSRNGKTSKKI	1.98	5.67	4.42
2ebn	136	147	YQTPPPSGFVTP	1.72	7.88	3.21
2exo	293	304	LVWDASYAKKPA	2.27	5.46	4.61
2pgd	361	372	WRGGCIIRSVFL	1.3	5	1.75
2sil	255	266	ETKDFGKTWTEF	1.46	5.52	2.09
2tgi	48	59	CPYLWSSDTQHS	1.38	3.89	1.57
3hsc	72	83	RLIGRRFDDAVV	1.18	4.95	1.18
451c	16	27	HAIDTKMVGPAY	1.69	6.39	6.82
4enl	372	383	SHRSGETEDTFI	1.22	2.83	2.32
4i1b	46	57	FVQGEESNDKIP	2.29	7.3	3.98
Mean				**1.53**	**5.23**	**2.99**

R_{min}, R_{ave} and R_{Emin} denote the average minimum backbone RMSD, the average ensemble RMSD and the average RMSD of the lowest energy conformations of the 1,000 loop ensemble with the same length, respectively.

Table 13: **The average RMSD of lowest energy conformations (R_{Emin}) of 8-residue loops on Test Set 4, obtained by using LOOPY, LOOPY/PLOP, DFIRE, LoopBuilder and DiSGro.**

PDB	Range	Sequence	LOOPY	LOOPY/PLOP	DFIRE	LoopBuilder	DiSGro
135l	84-91	LSSDITAS	1.46	2.61	0.40	0.40	0.83
1a3c	92-99	IPVDITDQ	3.36	2.06	1.29	3.31	1.98
1a621	71-78	SYLAGPDD	4.95	4.33	2.23	1.59	2.13
1a622	103-110	PPKEGERY	3.84	2.00	2.35	3.05	1.7
1ads	274-281	KVFDFELS	1.05	1.09	0.78	0.38	1.24
1alc	34-41	SGYDTQAI	3.06	6.51	0.82	6.45	0.84
1amm2	81-88	IPQHTGTF	2.00	1.27	2.47	2.13	1.95
1amm3	158-165	GAMNAKVG	1.14	2.37	1.24	0.36	2.42
1arb1	136-143	RRDQNYPG	1.93	1.65	2.88	0.46	1.38
1arb2	212-219	GPSSCSAT	1.63	0.44	1.30	0.77	1.97
1arb3	249-256	DPASTGAQ	4.06	2.11	1.79	2.24	1.03
1aru	234-241	LSPFPGEF	1.14	0.84	0.79	0.75	1
1btk1	67-74	VVPEKNPP	2.11	1.90	1.58	1.41	2.11
1btk2	133-140	RYNSDLVQ	3.73	4.86	4.35	1.54	1.4
1btl	50-57	DLNSGKIL	1.56	0.53	1.40	1.81	1.77
1c52	97-104	VKGFKPFT	1.78	1.65	0.80	1.19	1.32
1cex	73-80	VGGAYRAT	1.53	4.41	3.14	0.85	3.92
1clc	313-320	FRPYDPQY	0.77	2.09	1.55	0.31	1.13
1cvl1	148-155	TLVSSSHN	1.58	1.09	2.50	1.46	1.17
1cvl2	229-236	TSTGTLDV	2.10	2.08	2.01	0.67	1.96
1ddt	127-134	FGDGASRV	2.08	1.79	2.39	0.86	1.32
1ezm1	92-99	GTSPLTHK	2.04	2.09	3.05	0.33	1.16
1fnd	262-269	LKKDNTYV	0.66	0.90	2.12	0.52	0.47
1gof	606-613	VPSDSGVA	0.88	0.53	0.65	0.51	0.69
1hbq	31-38	DPEGLFLQ	1.36	1.72	2.64	1.27	1.14
1iab	48-55	RTTESDYV	0.73	2.42	3.25	1.83	2.57
1lit	82-89	DPKKNRRW	6.11	2.20	2.74	2.02	1.63
1lst	101-108	PIQPTLES	1.78	5.02	3.96	0.73	2.03
1mpp	74-81	TYGTGGAN	2.91	1.24	1.18	1.25	1.56
1mrp	68-75	AGLLAPIS	0.49	0.30	0.49	1.61	1.32
1msi	26-33	VVTPVGIP	1.44	2.53	1.18	1.01	1.25
1nar	192-199	FSNQQKPV	1.28	0.68	1.13	0.64	1.17
1nfp	118-125	NVDIANVR	1.24	3.69	1.22	0.97	1.49
1nif1	221-228	NGAVGALT	2.57	1.26	1.17	1.10	2.16
1nif2	279-286	ETWLIPGG	0.81	0.50	0.62	0.50	1.04
1nls	97-104	TGLYKETN	0.83	0.93	0.75	0.21	1.26
1nwp	84-91	TKVIGAGE	1.81	0.79	0.79	0.20	0.82
1oyc	80-87	GGYDNAPG	1.57	0.73	0.74	0.56	0.94
1poa	71-78	CSQGTLTC	1.66	1.44	1.15	0.89	2.03

1ppn2	191-198	RGTGNSYG	3.12	1.60	3.26	3.63	2.45
1prn	150-157	DPDQTVDS	3.16	2.26	1.17	0.78	2.16
1rro	18-25	CQDPDTFE	1.22	0.64	2.80	0.69	1.13
1sbp	107-114	KQIHDWND	0.69	0.33	1.15	0.30	1.82
1thw	18-25	SKGDAALD	1.70	1.87	1.58	0.34	1.23
1tml	187-194	NTSNYRWT	1.48	2.14	1.31	0.72	0.58
1wer1	824-831	SKQSCELS	1.59	0.49	1.64	1.35	0.67
1wer2	916-923	NIISDSPS	1.36	2.20	0.72	1.85	1.52
1xnb	99-106	KSDGGTYD	1.34	5.05	2.57	1.32	2.21
2arc	28-35	ANGYLDFF	2.51	0.98	1.14	1.29	3.78
2ayh	123-130	YTNGVGGH	1.70	3.35	1.52	1.01	0.7
2ayh1	124-131	TNGVGGHE	1.01	1.69	1.65	3.50	1.22
2ayh2	194-201	GSYNGANP	2.27	1.54	1.59	1.60	1.82
2cmd	270-277	LGKNGVEE	1.40	4.96	1.99	0.36	1.48
2ctc	53-60	STGGSNRP	2.37	3.31	1.71	0.99	2.41
2exo	262-269	MQVTRCQG	0.48	0.55	0.53	0.38	1.15
2sga	32-43	TTGGSRCS	1.53	0.95	1.22	0.97	1.41
3nul	36-43	SAKFPQLK	1.63	1.18	1.11	0.72	2.11
3seb	40-47	SIDQFLYF	1.15	0.27	0.71	0.40	0.92
5p21	45-52	VIDGETCL	1.21	0.61	0.93	0.56	1.97
5ptp1	22-29	CGANTVPY	3.42	3.12	2.37	3.98	1.35
5ptp2	172-179	YPGQITSN	0.57	2.02	0.96	1.30	2.83
7rsa	64-71	ACKNGQTN	2.09	2.87	3.49	3.04	1.71
8dfr	65-72	RPLKDRIN	2.91	2.69	2.21	3.00	2.4
Mean			1.89	1.96	1.69	1.31	**1.59**

The results of all the methods except DISGRO are obtained from Table A1 in Ref. [1].

Table 14: **The average RMSD of lowest energy conformations (R_{Emin}) of 9-residue loops on Test Set 4, obtained by using LOOPY, LOOPY/PLOP, DFIRE, LoopBuilder and DiSGro.**

PDB	Range	Sequence	LOOPY	LOOPY/PLOP	DFIRE	LoopBuilder	DiSGro
1aac	58-66	VAGVLGEAA	3.97	7.17	1.65	1.62	2.51
1aba	69-77	FAPDGSHIG	1.50	0.76	0.91	0.90	1.33
1amp	57-65	SASLPNASV	1.41	4.92	1.14	0.77	1.83
1arb1	90-98	ANGDGSMSQ	0.96	3.60	1.22	0.55	1.16
1arb2	168-176	AWGGGAGTT	6.07	7.84	6.54	6.58	1.68
1arp	127-135	SNCPGSPRL	0.57	0.24	0.45	0.28	1.61
1aru	36-44	NFYQGSKCE	2.67	7.59	2.06	1.30	2.52
1btl	102-110	LVEYSPVTE	6.22	1.08	1.96	3.37	2.55
1byb	246-254	GFFKSNGTY	2.23	5.61	1.14	0.79	1.31
1cse	95-103	VLNSSGSGS	2.81	7.02	0.39	0.62	2.49
1csh	252-260	GSALSDPYL	1.92	5.49	1.22	1.01	1.1
1ede	257-265	GMKDKLLGP	1.59	3.37	1.24	0.76	1.83
1fus	91-99	THTGASGNN	3.59	5.22	7.39	1.90	3.3
1fus1	31-39	DTAGSSTYP	2.90	5.95	4.33	4.00	2.92
1gpr	63-71	VFPTKHAIG	2.04	2.07	3.92	1.90	2.73
1isu	30-38	PGASPTAAG	6.18	4.99	4.22	6.16	3.13
1ivd	244-252	GSASGRADT	5.48	4.45	4.94	4.37	2.55
1lkk1	142-150	LAPGNTHGS	1.79	0.79	2.52	0.86	0.98
1lkk2	193-201	ISPRITFPG	2.32	0.73	1.34	0.91	1.88
1mla	194-202	LPVSVPSHC	2.07	3.77	3.87	4.48	2.16
1mrj	92-100	AKYVFKDAM	2.79	5.89	1.55	2.04	2.05
1mrk	53-61	TNYADETIS	1.22	4.06	1.30	0.63	1.81
1mrp	284-292	EAPVVSATT	2.04	1.48	2.86	2.74	1.51
1nfp	12-20	NFYHVGQQE	1.74	4.60	3.88	3.79	5.12
1nif	266-274	ATGKFRNPP	1.11	1.31	1.97	0.57	1.4
1nls	131-139	NQFSKDQKD	3.55	4.92	3.14	3.86	2.03
1noa	76-84	FLFDGTRWG	4.61	7.97	2.95	2.51	0.89
1noa1	9-17	PSSGLSDGT	5.99	7.72	5.43	6.00	1.65
1noa2	99-107	DAAGNGPEG	4.90	2.55	4.90	3.34	1.86
1npk	102-110	ASAPGSIRG	1.27	0.30	0.74	0.23	0.82
1onc	70-78	VTSRPCKYK	10.31	7.52	3.01	1.70	1.69
1pda	108-116	FVSNNYDSL	1.28	0.91	0.98	0.58	1.24
1pgs	117-125	TETWLAKGR	0.72	1.84	2.13	0.59	2.14
1php	91-99	TNEAVGDEV	2.45	1.37	2.43	0.60	0.48
1ptf	10-18	AETGIHARP	3.23	3.02	1.51	2.12	1.38
1ra9	142-150	DADAQNSHS	2.30	2.72	3.39	1.99	2.5
1rhs	216-224	LTEDGFEKS	2.70	5.62	2.91	1.54	2.5
1sgp	109-117	TNTTIPKDG	1.51	2.31	2.29	0.63	2.31
1tca1	170-178	AGGLTQIVP	2.04	0.99	1.76	0.47	1.24

1tca2	217-225	GPLFVIDHA	1.95	0.48	1.58	0.47	1.22
1wer	942-950	NLVEFGAKE	5.66	7.29	8.31	5.34	1.81
1xif	59-67	IPFGSSDSE	3.71	5.59	2.99	1.04	1.65
1xnb	133-141	QSKRPTGSN	1.06	0.87	1.95	1.02	2.05
1xnb1	116-124	PSIDGDRTT	3.58	0.97	1.15	1.76	2.02
1xyz1	568-576	QPRQNVFDF	1.74	4.86	1.83	1.01	1.07
1xyz2	795-803	WGFTDKYTW	1.67	0.98	1.80	2.55	1.16
2ayh	169-177	TANIPSTPG	0.74	1.06	2.32	0.55	2.13
2cpl	24-32	LFADKVPKT	2.72	5.65	1.00	0.22	1.00
2eng	172-180	DWFKNADNP	1.81	1.93	1.65	2.14	0.97
2hbg	18-26	AGADNGAGV	1.40	4.18	1.78	0.43	1.09
2sil	183-191	QLNDGKLVF	1.74	2.72	1.32	0.33	2.24
3pte1	78-86	EGKLDLDAS	0.96	0.46	0.96	0.51	0.85
3pte2	107-115	SHRSGLYDY	1.20	0.37	1.52	0.33	2.18
3pte3	215-223	TPDEAGGAL	4.19	4.28	3.87	3.29	0.59
3tgl	56-64	STLIYDTNA	1.61	6.15	3.27	3.12	2.35
4gcr	94-102	ERDDFRGQM	1.74	7.81	2.11	1.99	2.18
Mean			2.71	3.67	2.52	1.88	**1.83**

The results of all the methods except DISGRO are obtained from Table A1 in Ref. [1].

Table 15: **The average RMSD of lowest energy conformations (R_{Emin}) of 10-residue loops on Test Set 4, obtained by using LOOPY, LOOPY/PLOP, DFIRE, LoopBuilder and DISGRO.**

PDB	Range	Sequence	LOOPY	LOOPY/PLOP	DFIRE	LoopBuilder	DISGRO
135l	18-27	DNYRGYSLGN	1.53	8.30	1.55	1.12	1.86
1ads	171-180	NKPGLKYKPA	1.06	0.50	1.50	0.59	2.14
1ads1	170-179	LNKPGLKYKP	0.96	1.29	5.05	0.68	1.6
1amp	181-190	TNYKGSAQDV	2.30	1.80	2.78	2.21	1.53
1arb	41-50	VNNTANDRKM	1.76	3.16	0.88	1.37	1.86
1arp	37-46	FYQGSKCESP	2.16	4.52	1.45	0.86	1.51
1aru	128-137	NCPGSPRLEF	2.21	0.25	2.63	0.23	0.91
1avm	145-154	DHQNNLPAGS	3.54	4.07	3.36	3.58	2.03
1btl	170-179	NEAIPNDERD	1.84	3.38	2.34	2.57	1.72
1dim1	87-96	YNDRVNSKLS	2.98	NA	2.21	2.18	2.37
1dim2	131-140	YRDKAPDTDW	1.27	3.35	2.10	2.18	1.64
1ede	164-173	FVTQPADGFT	4.19	6.86	4.21	0.92	1.99
1edg	269-278	GGTNAWNIND	1.15	1.95	0.60	0.25	0.73
1ezm	237-246	ANSPGWDTRK	0.56	0.75	0.67	0.42	1.27
1fkf	63-72	VAQMSVGQRA	0.83	0.42	0.56	0.26	1.54
1gpr	133-142	FTNLAEGETV	3.43	2.83	4.64	4.65	2.09
1gvp	49-58	LDEGQPAYAP	2.79	1.54	2.73	3.03	1.7
1ixh	84-93	NIPGLKSGEL	4.67	3.63	1.57	0.39	2.74
1knt	35-44	YGGCGGNENK	1.48	5.24	1.66	4.32	2.23
1mrj	173-182	KRVDKTFLPS	2.85	7.86	0.77	0.36	1.03
1onc	48-57	CKGIIASKNV	3.81	2.41	5.66	5.02	3.61
1pgs	68-77	KNKTTGEWYE	2.30	0.76	2.74	0.74	1.25
1plc	42-51	DEDSIPSGVD	5.38	4.28	1.35	2.30	2.16
1ppn	190-199	KRGTGNSYGV	2.12	3.10	2.64	1.73	2.67
1scs	65-74	VSYPNADSAT	2.00	6.34	2.57	1.48	1.56
1tca1	23-32	QGASPSSVSK	5.90	5.97	1.97	0.89	1.69
1tca2	258-267	CNPLPANDLT	1.77	0.28	0.89	0.22	2.19
1whi	47-56	TPGGVVKKGQ	2.15	5.70	1.46	1.56	1.46
2alp	90-105	RVFPGNDRAW	0.70	NA	1.61	2.13	1.56
2ayh	80-89	KPAKNTGIVS	1.48	1.33	0.63	0.27	1.5
2cmd	57-66	GFSGEDATPA	4.26	7.42	0.85	2.27	0.45
2mnr	91-100	FCLAGYTGLI	4.14	3.47	2.91	2.99	1.43
2sil	197-206	RTKNITTVLN	1.60	1.87	4.76	2.74	1.6
3hsc	28-37	IIANDQGNRT	3.35	4.47	3.95	4.40	2.75
3seb	200-209	MMPAPGDKFD	0.89	0.34	0.54	0.28	1.3
3tgl	257-266	HLSYFGINTG	1.52	1.71	1.00	0.94	1.7
4fgf	42-51	GVREKSDPHI	2.53	4.76	3.41	3.31	0.68
7rsa	110-119	CEGNPYVPVH	2.32	2.96	2.91	2.33	2.43
7rsa1	33-42	RNLTKDRCKP	2.65	4.49	3.64	3.54	3.45

7rsa2	87-96	TGSSKYPNCA	3.16	6.32	5.21	6.29	3.22
Mean			2.42	3.40	2.41	1.93	**1.83**

The results of all the methods except DISGRO are obtained from Table A1 in Ref. [1].

Table 16: **The average RMSD of lowest energy conformations (R_{Emin}) of 11-residue loops on Test Set 4, obtained by using LOOPY, LOOPY/PLOP, DFIRE, LoopBuilder and DISGRO.**

PDB	Range	Sequence	LOOPY	LOOPY/PLOP	DFIRE	LoopBuilder	DISGRO
153l	154-164	VRSYARMDIGT	2.41	3.59	3.32	0.80	2.26
1a2p	76-86	INYTSGFRNSD	2.13	9.30	3.05	2.68	1.78
1a2y	91-101	FWSTPRTFGGG	2.39	2.07	2.78	1.44	2.29
1a8d	195-205	ITGLGAIREDN	1.97	2.45	1.64	1.51	1.79
1ads	290-300	SYNRNWRVCAL	1.71	1.54	2.22	1.24	1.51
1ako	110-120	FPQGESRDHPI	3.05	2.11	7.88	1.89	1.97
1akz	211-221	AHQANSHKERG	2.56	3.05	1.23	1.16	2.75
1aol	152-162	VYWKPSSSWDY	3.01	3.94	3.26	2.86	1.41
1aru	297-307	IPSAVSNNAAP	2.38	1.67	2.36	2.40	1.86
1awq	1101-1111	ANAGPNTNGSQ	2.03	1.67	2.11	0.77	1.38
1bol	33-43	WAPGYGPDNAF	1.84	3.82	2.83	4.20	2.78
1bx4	250-260	ALPKMNSKRQR	2.42	2.29	4.82	0.94	2.42
1c5e	82-92	YEDVLWPEAAS	2.36	9.04	2.70	0.37	2.12
1cb0	33-43	YVDTPFGKPSD	8.16	4.22	2.21	2.91	1.35
1cnv	162-172	APGCLSPDEYL	3.88	2.47	4.05	1.88	1.92
1cs6	145-155	NEFPNFIPADG	4.31	7.95	3.68	5.11	2.58
1cvl	257-267	RASGQNDGLVS	2.81	11.56	1.54	1.32	7.88
1dad	42-52	GSEKTPEGLRN	4.49	4.12	13.49	3.14	3.71
1dys	290-300	FGQPFTTNTNN	2.37	10.06	2.23	1.90	2
1edt	93-103	NHQGAGFANFP	1.93	1.62	1.49	0.55	1.35
1eur	87-97	RPTGIDAPGPN	2.81	5.95	5.26	4.14	3.1
1exm	291-301	RGVSREEVERG	3.53	4.09	2.53	1.47	0.75
1f46	64-74	MVKPGTFDPEM	5.06	4.18	1.78	2.23	2.53
1fus	28-38	QNDDTAGSSTY	1.87	8.25	6.37	0.38	2.59
1g6s	77-87	GNGGPLHAEGA	2.92	3.94	1.04	1.45	0.91
1g9g	125-135	LDTSQPVGRDP	2.43	2.30	1.01	2.67	1.89
1gmu	44-54	LPRGLLLRGGD	3.76	3.14	4.51	1.20	1.62
1gqv	114-124	RDQRRDPPQYP	2.79	6.46	6.38	3.03	2.38
1i4j	86-96	LPRARGRADII	4.18	4.61	8.38	4.59	1.73
1iu8	85-95	ARIPDNDGEQP	2.00	9.05	9.53	4.54	1.1
1ixh	120-130	NPGLKLPSQNI	2.04	1.44	1.81	1.13	1.45
1jp4	179-189	GFQLKEAPAGK	2.20	4.05	1.39	2.04	2.38
1k7i	137-147	TRDASGNLDYG	3.68	8.11	5.57	7.87	4.41
1lmi	34-44	STAVIPGYPVA	2.54	2.64	1.76	0.90	1.68
1mla	9-19	PGQGSQTVGML	4.13	1.84	4.68	3.60	4.06
1my7	254-264	TPPYADPSLQA	2.27	1.89	2.85	2.00	1.36
1nln	26-36	DKRFPGFVSPH	1.15	1.48	1.08	0.91	1.8
1nls	216-226	NIDSSIPSGST	3.95	1.64	1.66	3.45	1.88
1nog	98-108	GKIELFVVPGG	2.69	2.05	2.81	2.53	2.39

1nsc	383-393	DGDPWTDSDAL	4.51	8.59	3.61	4.87	3.51
1ojq	141-151	LVSGAALAGRP	5.12	5.92	3.43	5.35	1.4
1oth	69-79	QKGEYLPLLQG	1.68	2.44	1.21	1.38	2.15
1oyc	203-213	DPHSNTRTDEY	2.82	3.72	1.55	0.56	3.55
1pgs	83-93	TPYWVGTEKLP	1.92	3.39	3.24	4.08	2.52
1pkh	44-54	DDEVYDLSKEL	7.35	5.02	6.88	7.69	3.65
1qlw	31-41	ETLSLSPKYDA	1.91	11.88	1.98	1.82	3.53
1rcf	122-132	TDGYDFNDSKA	1.95	7.62	1.64	3.63	2.78
1t1d	127-137	SGGRLRRPVNV	2.33	0.89	3.35	0.66	3.25
1whi	25-35	LGGSGRRYANI	6.93	1.25	8.08	5.70	2.26
2eng	124-134	IPGGGVGIFDG	3.66	3.37	1.85	3.22	2.93
2pth	8-18	LANPGAEYAAT	2.69	3.50	2.32	1.28	2.55
2tgi	46-56	GACPYLWSSDT	1.86	4.67	1.88	0.95	2.73
3pte	91-101	LPGLLPDDRIT	1.97	2.45	2.09	1.78	1.74
5pti	7-17	EPPYTGPCKAR	2.11	4.99	2.65	3.07	3
Mean			3.02	4.36	3.43	2.50	**2.38**

The results of all the methods except DISGRO are obtained from Table A1 in Ref. [1].

Table 17: **The average RMSD of lowest energy conformations (R_{Emin}) of 12-residue loops on Test Set 4, obtained by using LOOPY, LOOPY/PLOP, DFIRE, LoopBuilder and DISGRO.**

PDB	Range	Sequence	LOOPY	LOOPY/PLOP	DFIRE	LoopBuilder	DISGRO
153l	98-109	KRSHKPQGTWNG	7.80	5.80	2.63	0.82	3.27
1a8d	155-166	DLPDKFNAYLAN	2.14	3.17	3.00	2.36	1.84
1akz	181-192	IEDFVHPGHGDL	1.73	3.26	2.78	3.55	2.15
1aoz	123-134	PPQGKKEPFHYD	2.74	4.99	2.40	4.24	2.34
1arb	74-85	NYQNSTCRAPNT	2.32	2.51	3.26	1.92	2.94
1arb	182-193	WQPSGGVTEPGS	2.97	3.07	1.62	3.12	1.38
1bhe	121-132	GQGGVKLQDKKV	3.37	4.12	3.37	1.00	1.26
1bkf	9-20	PGDGRTFPKRGQ	2.61	4.36	2.60	4.10	2.29
1bn8	298-309	STSSSSYPFSYA	2.14	4.78	1.70	0.72	4.26
1c5e	82-93	YEDVLWPEAASD	2.51	2.68	1.50	0.59	1.07
1cb0	33-44	YVDTPFGKPSDA	6.30	6.71	2.15	3.09	1.79
1cb8	327-338	DSTVAAGYKIEP	3.24	4.32	3.81	4.35	3.95
1cex	40-51	RGSTETGNLGTL	3.18	2.29	1.90	1.46	5.43
1cnv	188-199	FYNDRSCQYSTG	2.46	4.35	1.71	3.98	1.87
1cs6	145-156	NEFPNFIPADGR	4.93	9.14	3.74	4.06	2.47
1dim	213-224	TDGITWSLPSGY	0.68	0.67	1.94	2.10	0.97
1dqz	209-220	CGNGTPSDLGGD	2.72	1.68	7.60	2.21	1.81
1el5	275-286	IDPDTINREFGV	3.73	4.91	2.46	1.75	2.85
1exm	291-302	RGVSREEVERGQ	3.79	4.59	2.94	3.88	3.08
1f46	64-75	MVKPGTFDPEMK	2.72	2.54	2.46	2.31	2.91
1i7p	63-74	LPSPQHILGLPI	3.86	3.76	7.01	1.49	2.39
1ioo	179-190	RCPQSDTCDKTANA	2.04	NA	3.05	2.17	2.37
1ixh	160-171	VGTGSTVKWPIG	2.15	3.05	2.34	2.46	2
1iye	103-114	GDVGMGVNPPAGNA	4.31	NA	2.14	4.36	3.36
1jp4	179-190	GFQLKEAPAGKH	4.22	3.74	3.04	1.93	2.32
1kcm	118-129	PDLGTQENVHKL	2.65	5.61	2.28	2.79	2.05
1luc	158-169	LNPSAYTQGGAP	2.75	4.05	3.60	3.85	4.25
1m3s	68-79	VGEILTPPLAEG	6.15	6.36	6.03	5.89	5.03
1ms9	529-540	GSTPVTPTGSWE	1.53	1.75	1.99	1.67	2.1
1my7	254-265	TPPYADPSLQAP	2.27	1.65	3.64	1.91	1.39
1oth	69-80	QKGEYLPLLQGK	1.67	1.46	3.73	0.41	2.16
1oyc	203-214	DPHSNTRTDEYG	4.11	3.22	2.30	3.23	3.75
1pkh	44-55	DDEVYDLSKELN	5.29	11.46	5.58	4.42	3.23
1qlw	31-42	ETLSLSPKYDAH	2.33	6.39	4.52	2.95	2.97
1t1d	127-138	SGGRLRRPVNVP	2.27	1.22	2.97	2.13	2.51
1xyz	813-824	NPLIYDSNYNPK	1.05	6.77	1.05	0.65	2.68
2ayh1	21-32	ADGYSNGGVFNC	3.24	2.01	5.65	3.08	3.61
2hlc	91-102	HSMFNPDTYLND	3.88	5.09	4.29	5.44	3.56
2pia	30-41	DPQGAPLPPFEA	3.38	5.21	1.14	3.35	1.25

2ptd	136-147	YFVDPIFLKTEG	2.77	5.20	1.71	1.42	1.84
Mean			3.15	4.11	3.15	2.65	**2.62**

The results of all the methods except DISGRO are obtained from Table A1 in Ref. [1].

Table 18: **The average RMSD of lowest energy conformations (R_{Emin}) of 13-residue loops on Test Set 4, obtained by using LOOPY, LOOPY/PLOP, DFIRE, LoopBuilder and DISGRO.**

PDB	Range	Sequence	LOOPY	LOOPY/PLOP	DFIRE	LoopBuilder	DISGRO
16pk	132-144	ENVRFYKEEGSKK	3.28	6.48	4.12	2.40	4.49
1a8d	155-167	DLPDKFNAYLANK	3.36	4.77	2.01	1.71	3.15
1a8d1	74-86	KAMDIEYNDMFNN	3.12	4.49	3.09	6.53	3.79
1arb	182-194	WQPSGGVTEPGSS	2.87	3.43	2.71	3.21	1.95
1bhe	121-133	GQGGVKLQDKKVS	10.18	9.63	6.31	11.02	3.91
1bkp	51-63	DNSEVPILTTKKV	2.37	1.11	1.30	0.82	3.91
1cnv	110-122	LSERREGPLGKVA	10.47	10.18	2.84	12.52	1.56
1cru	357-369	YKGGKKAITGWEN	3.90	6.75	9.68	3.58	4.89
1dpg	352-364	KAGTFNFGSEQEA	5.26	6.68	2.08	1.37	8.97
1dqz	209-221	CGNGTPSDLGGDN	7.72	5.77	6.98	2.40	2.68
1dys	290-302	FGQPFTTNTNNPN	3.17	1.45	3.77	2.42	1.46
1ed8	67-79	GAGGFFKGIDALP	3.24	5.02	1.02	0.51	3.03
1eok	147-159	GYNGWYSGSMAAT	2.78	2.02	3.84	0.71	1.67
1f46	64-76	MVKPGTFDPEMKD	3.03	3.27	3.54	2.75	2.21
1g8f	72-84	SRLADGTLWTIPI	6.55	7.20	2.88	6.07	1.85
1gpi	308-320	NSVANIPGVDPVN	2.98	11.88	2.07	0.90	4.49
1hnj	191-203	LPNADRVNPENSI	10.00	5.49	10.39	10.34	3.33
1hxh	87-99	NAGILLPGDMETG	7.70	8.15	2.09	0.61	7.48
1iir	197-209	AADPVLAPLQPTD	4.54	4.08	5.45	10.62	1.46
1jp4	153-165	PYYNYQAGPDAVL	6.18	5.18	3.61	7.13	2.03
1kbl	793-805	KIYESDPFARLDQ	3.33	6.81	3.29	1.21	3.15
1krh	131-143	DDGQPDIHFLAGQ	3.41	6.07	2.16	0.40	2.84
1l8a	691-703	TLNENYHMPAMPE	1.35	3.97	2.38	2.37	0.44
1lki	62-74	PNMTDFPSFHGNG	1.95	8.48	8.69	6.28	2.5
1m3s	68-80	VGEILTPPLAEGD	5.79	1.62	4.01	1.55	5.1
1mo9	107-119	SGQYWFPDMTEKV	3.29	12.29	2.38	2.03	4.52
1nln	26-38	DKRFPGFVSPHKL	2.01	8.00	4.44	5.90	1.65
1o6l	386-398	KKDPKQRLGGGPS	6.48	8.57	4.10	2.24	5.77
1ock	43-55	DKSARAQASGPLR	6.96	2.77	5.28	3.97	1.53
1ojq	167-179	IDSKELTAYPGQQ	2.07	4.70	3.65	4.24	5.05
1os8	141-153	WGANREGGSQQR	4.80	10.21	6.14	3.37	2.69
1p1m	327-339	FKSGKIEEGWNAD	2.07	5.28	3.26	0.99	2.15
1qqp	161-173	PFVGVNRYDQYKV	2.19	7.08	2.74	2.96	1.10
1qsl	389-401	YLDAPDQISRERA	3.54	5.60	3.59	2.03	4.55
1xyz	645-657	ECMDDSGNGLRSS	4.81	5.86	11.60	3.29	4.54
1yge	452-464	LPHSAGDLSAAVS	4.16	5.86	11.60	3.29	2.8
2hlc	91-103	HSMFNPDTYLNDV	3.08	5.91	4.29	3.63	4.6
2olb	207-219	NPQYWDNAKTVIN	3.50	4.20	4.15	3.38	2.88
2ptd	136-148	YFVDPIFLKTEGN	5.91	4.00	1.91	1.48	2.32

3grs	404-416	TPMYHAVTKRKTK	4.22	3.43	4.60	7.17	2.00
Mean			4.44	5.84	4.35	3.74	**3.26**

The results of all the methods except DISGRO are obtained from Table A1 in Ref. [1].

Table 19: **Results of 14-residue loops on Test Set 5, obtained by using PLOP [2] and DISGRO.**

Target	PDB	Start	End	Sequence	R_{Emin}(PLOP)	R_{min}(DISGRO)	R_{Emin}(DISGRO)
1	1E6U	A:274	A:287	ASKPDGTPRKLLDV	1.94	1.50	3.92
2	1JP4	A:153	A:166	PYYNYQAGPDAVLG	7.26	1.67	2.50
3	1N0Q	A:24	A:37	AGADVNAKDKNGRT	0.22	1.30	4.93
4	1N0Q	A:57	A:70	AGADVNAKDKNGRT	0.52	1.29	2.43
5	1O97	D:156	D:169	RPSVFKPLEGAGSP	0.82	1.36	1.72
6	1OCK	A:209	A:222	GRSEFSGIVPAKAP	0.93	1.54	2.07
7	1P3C	A:112	A:125	GYRSIRQVTNLTGT	0.32	0.96	2.16
8	1P3D	A:402	A:415	DVYAAGEAPIVGAD	1.74	1.73	2.39
9	1R6X	A:72	A:85	SRLADGTLWTIPIT	0.30	1.99	3.05
10	1RDQ	E:273	E:286	LQVDLTKRFGNLKN	1.50	1.90	4.37
11	1RV9	A:225	A:238	GTHCTVLERDTFFS	0.26	0.71	1.09
12	1VYR	A:193	A:206	SPSSNQRTDQYGGS	0.58	2.45	4.97
13	1VYR	A:235	A:248	SPIGTFQNVDNGPN	1.17	1.74	2.95
14	1XU1	A:221	A:234	PRANAKLSLSPHGT	0.47	1.19	1.90
15	1ZEQ	X:53	X:66	ITPQTKMSEIKTGD	0.27	1.67	5.28
16	2BWR	A:269	A:282	KDFGVNSGWRVEKH	0.44	1.37	4.98
17	2BWR	B:158	B:171	NNFGYAQGWRLDRH	4.56	2.22	3.61
18	2C0H	A:40	A:53	QAWVNYARDFGHNQ	5.96	2.19	6.74
19	2EX2	A:139	A:152	TSIFASHDKAPGWP	0.21	1.59	4.29
20	2GGC	A:79	A:92	HGIPDDAKLLKDGD	0.24	1.69	4.84
21	2H3L	A:1360	A:1373	QPEGPASKLLQPGD	0.28	1.21	2.32
22	2O2K	A:1221	A:1234	SNLKSKYFAVGKIS	1.07	1.44	3.06
23	2PVQ	A:139	A:152	LSDKNAYWLGDDFT	0.66	1.44	1.44
24	2VFR	A:325	A:338	AADAQWLSPAYGRD	0.34	1.90	2.71
25	3B40	A:389	A:402	SDFNDGGGVDGWKD	1.28	1.95	6.37
26	3B64	A:44	A:57	DSTPMHFFGSTDPV	0.65	2.06	4.27
27	3BY9	A:177	A:190	DLSAIEQGWQNKSS	1.13	1.83	4.01
28	3BY9	A:205	A:218	SQPAWLFHSVADLS	0.28	1.45	3.32
29	3CFZ	A:125	A:138	TDKSKYKDEINSTN	0.68	1.36	5.80
30	3CNQ	S:50	S:63	FVPSETNPFQDNNS	1.03	1.13	8.37
31	3CSS	A:163	A:176	FGSDGHTASIFPDS	0.21	1.11	3.19
32	3DRF	A:550	A:563	KRVVGMTLDYGAMN	1.85	2.04	3.70
33	3E7H	A:67	A:80	GKVSAKDYVNEATG	1.78	1.23	3.49
34	3EHR	A:95	A:108	NRVGVNGLDKAGST	0.94	2.03	5.52
35	3FOT	A:164	A:177	DVSTDSTPIPQDAT	0.30	1.18	2.21
36	3HXL	A:277	A:290	ARVNESLTYQGYDE	0.79	1.60	4.51
Mean					1.19	**1.58**	**3.73**

R_{min} and R_{Emin} are the minimum backbone RMSD and average RMSD of the lowest energy conformations, respectively.

Table 20: **Results of 15-residue loops on Test Set 5, obtained by using PLOP [2] and DISGRO.**

Target	PDB	Start	End	Sequence	R_{Emin}(PLOP)	R_{min}(DISGRO)	R_{Emin}(DISGRO
1	1AH7	A:157	A:171	KVTDGNGYWNWKGTN	0.32	1.21	1.73
2	1BHE	A:121	A:135	GQGGVKLQDKKVSWW	0.42	1.73	5.96
3	1H4A	X:19	X:33	SSDHPNLQPYLSRCN	0.28	1.58	4.62
4	1JU3	A:486	A:500	RETLVNPTLIEAGEI	0.35	1.96	1.96
5	1QAZ	A:298	A:312	DKSARAQASGPLRGI	1.68	1.86	5.11
6	1QQF	A:1112	A:1126	QKPDGVFQEDGPVIH	0.31	1.57	2.58
7	1RA0	A:283	A:297	QGRFDTYPKRRGITR	2.78	2.22	3.88
8	1RA0	A:361	A:375	LNLQDYGIAAGNSAN	0.39	1.64	2.78
9	1RYO	A:172	A:186	QLCPGCGCSTLNQYF	0.88	1.21	2.4
10	1S95	A:477	A:491	TAVPHPNVKPMAYAN	0.61	2.07	3.45
11	1WB4	A:1033	A:1047	ALPHFDYTSDFSKGN	0.21	2.47	6.14
12	1WUI	L:454	L:468	KGDNVICAPWEMPKQ	1.81	1.18	2.46
13	1Y12	A:10	A:24	GDVKGESKDKTHAEE	0.36	2.16	11.73
14	1ZHX	A:392	A:406	NLSTKNAPSGTLVGD	7.10	1.92	4.8
15	2AEB	B:156	B:170	IPDVPGFSWVTPCIS	2.55	1.54	3.22
16	2B0T	A:701	A:715	VQGGATDLGGYYSPN	1.23	1.94	3.17
17	2CJP	A:58	A:72	DLRGYGDTTGAPLND	0.46	1.22	5.63
18	2DSJ	A:354	A:368	GGGRKRKGEPIDHGV	0.51	1.56	2.96
19	2H3L	A:1339	A:1353	GVGGRGNPFRPDDDG	1.10	2.29	2.66
20	2O2K	A:1220	A:1234	FSNLKSKYFAVGKIS	1.36	1.51	2.06
21	2OIT	A:290	A:304	FMEPCYGSCTERQHH	0.54	1.75	5.17
22	2PKF	A:26	A:40	LPEHLHKVSLSFLVD	2.34	2.49	4.44
23	2V3V	A:382	A:396	WGLPEGRIAPEPGYH	0.35	1.53	1.75
24	3A3P	A:286	A:300	DSNDNIASFSNRQPE	0.18	1.77	2.63
25	3A64	A:350	A:364	SPASHVPAPEAGEWF	2.55	2.37	5.53
26	3BB7	A:231	A:245	SEMQYGGPNEGSGAY	6.26	1.61	2.72
27	3BF7	A:49	A:63	DVRNHGLSPREPVMN	5.66	1.72	5.34
28	3CSS	A:95	A:109	LLRDVPSSDVISIDR	2.36	2.3	4.41
29	3EA1	A:136	A:150	YFVDPIFLKTEGNIK	0.49	1.91	2.21
30	3F1L	A:99	A:113	NAGLLGDVCPMSEQN	1.05	1.66	3.68
Mean					1.55	**1.80**	**3.91**

R_{min} and R_{Emin} are the minimum backbone RMSD and average RMSD of the lowest energy conformations, respectively.

Table 21: **Results of 16-residue loops on Test Set 5, obtained by using PLOP [2] and DISGRO.**

Target	PDB	Start	End	Sequence	R_{Emin}(PLOP)	R_{min}(DISGRO)	R_{Emin}(DISGRO)
1	1C1K	A:31	A:46	NGKYDVIKYNWCMRVS	0.66	1.62	2.3
2	1DJ0	B:19	B:34	DGSKYYGWQRQNEVRS	7.08	2.42	5.68
3	1GPI	A:308	A:323	NSVANIPGVDPVNSIT	0.33	2.06	5.55
4	1UG6	A:340	A:355	GAAYPDLWTGEAVVED	0.43	1.37	3.25
5	1WHI	A:88	A:103	RDDKSPRGTRIFGPVA	0.66	1.97	2.68
6	1WM3	A:67	A:82	INETDTPAQLEMEDED	0.32	2.46	3.29
7	1ZHV	A:20	A:35	SASEAIPAWADGGGFV	0.64	1.92	5.91
8	2BG1	A:708	A:723	SPSIWGNERFALDPSV	2.15	2.11	3.7
9	2GGC	A:184	A:199	LHYDSRETNVVLKPGM	1.08	1.25	3.82
10	2HKJ	A:418	A:433	TKIPYKSAGKESIAEV	0.42	1.82	5.29
11	2PKF	B:25	B:40	LLPEHLHKVSLSFLVD	0.93	1.94	3.5
12	2PUH	A:70	A:85	DSPGFNKSDAVVMDEQ	1.52	1.56	6.62
13	2PYW	A:321	A:336	NKDGPGEAYLADIYNN	2.57	1.9	4.76
14	3IFE	A:14	A:29	KIDTQSNEDSHTVPTT	1.18	1.99	1.99
Mean					1.43	**1.88**	**4.16**

R_{min} and R_{Emin} are the minimum backbone RMSD and average RMSD of the lowest energy conformations, respectively.

Table 22: **Results of 17-residue loops on Test Set 5, obtained by using PLOP [2] and DISGRO.**

Target	PDB	Start	End	Sequence	R_{Emin}(PLOP)	R_{min}(DISGRO)	R_{Emin}(DISGRO)
1	1KWG	A:314	A:330	QPGPVNWAPHNPSPAPG	1.93	1.86	3.89
2	1QLW	A:145	A:161	FRFGPRYPDAFKDTQFP	0.41	2.51	5.13
3	1VJU	A:277	A:293	LPPRARWGYNWQPEPGT	0.63	1.34	3.34
4	2FAO	A:814	A:830	AGDDPWADYAGTRQRIS	0.50	2.41	4.41
5	2HDW	A:131	A:147	DPSYTGESGGQPRNVAS	2.22	1.32	4.94
6	2PEF	A:191	A:207	TNGMIDKILNKIDPEDV	1.92	1.78	1.86
7	3A3P	A:262	A:278	SGNEGAPSPSYPAAYPE	0.52	2.31	7.34
8	3H2G	A:124	A:140	DYLGLGKSNYAYHPYLH	4.07	2.46	4.77
9	3HUH	A:71	A:87	QEMEFEPKASRPTPGSA	8.56	3.6	4.51
Mean					2.30	**2.18**	**4.46**

R_{min} and R_{Emin} are the minimum backbone RMSD and average RMSD of the lowest energy conformations, respectively.

References

[1] C.S. Soto, M. Fasnacht, J. Zhu, L. Forrest, and B. Honig. Loop modeling: Sampling, filtering, and scoring. *Proteins: Structure, Function, and Bioinformatics*, 70(3):834–843, 2008.

[2] S. Zhao, K. Zhu, J. Li, and R.A. Friesner. Progress in super long loop prediction. *Proteins: Structure, Function, and Bioinformatics*, 2011.
